# Surface curvature-induced oriented assembly of sushi-like Janus therapeutic nanoplatform for combined chemodynamic therapy

**DOI:** 10.1186/s12951-023-02138-0

**Published:** 2023-11-15

**Authors:** Yanming Ma, Minchao Liu, Mengmeng Hou, Yufang Kou, Wenxing Wang, Tiancong Zhao, Xiaomin Li

**Affiliations:** Department of Chemistry, Laboratory of Advanced Materials, College of Chemistry and Materials, Shanghai Key Laboratory of Molecular Catalysis and Innovative Materials, State Key Laboratory of Molecular Engineering of Polymers, Collaborative Innovation Center of Chemistry for Energy Materials (2011-iChEM), Fudan University, Shanghai, 200433 China

**Keywords:** Mesoporous, Nanocatalytic medicine, Chemodynamic therapy, Janus nanoparticles, Asymmetric nanostructure

## Abstract

**Background:**

Chemodynamic therapy (CDT) based on Fenton/Fenton-like reaction has emerged as a promising cancer treatment strategy. Yet, the strong anti-oxidation property of tumor microenvironment (TME) caused by endogenous glutathione (GSH) still severely impedes the effectiveness of CDT. Traditional CDT nanoplatforms based on core@shell structure possess inherent interference of different subunits, thus hindering the overall therapeutic efficiency. Consequently, it is urgent to construct a novel structure with isolated functional units and GSH depletion capability to achieve desirable combined CDT therapeutic efficiency.

**Results:**

Herein, a surface curvature-induced oriented assembly strategy is proposed to synthesize a sushi-like novel Janus therapeutic nanoplatform which is composed of two functional units, a FeOOH nanospindle serving as CDT subunit and a mSiO_2_ nanorod serving as drug-loading subunit. The FeOOH CDT subunit is half covered by mSiO_2_ nanorod along its long axis, forming sushi-like structure. The FeOOH nanospindle is about 400 nm in length and 50 nm in diameter, and the mSiO_2_ nanorod is about 550 nm in length and 100 nm in diameter. The length and diameter of mSiO_2_ subunit can be tuned in a wide range while maintaining the sushi-like Janus structure, which is attributed to a Gibbs-free-energy-dominating surface curvature-induced oriented assembly process. In this Janus therapeutic nanoplatform, Fe^3+^ of FeOOH is firstly reduced to Fe^2+^ by endogenous GSH, the as-generated Fe^2+^ then effectively catalyzes overexpressed H_2_O_2_ in TME into highly lethal ·OH to achieve efficient CDT. The doxorubicin (DOX) loaded in the mSiO_2_ subunit can be released to achieve combined chemotherapy. Taking advantage of Fe^3+^-related GSH depletion, Fe^2+^-related enhanced ·OH generation, and DOX-induced chemotherapy, the as-synthesized nanoplatform possesses excellent therapeutic efficiency, in vitro eliminating efficiency of tumor cells is as high as ~ 87%. In vivo experiments also show the efficient inhibition of tumor, verifying the synthesized sushi-like Janus nanoparticles as a promising therapeutic nanoplatform.

**Conclusions:**

In general, our work provides a successful paradigm of constructing novel therapeutic nanoplatform to achieve efficient tumor inhibition.

**Supplementary Information:**

The online version contains supplementary material available at 10.1186/s12951-023-02138-0.

## Background

Tumor has always been a vital threat to human health [1, 2] and various methods have been developed for oncological treatment. Among them, methods utilizing nanomaterials to treat tumor have been receiving much research interests for their desirable therapeutic efficiency and minimized side effects [3–7]. Recently, treatments based on nanotechnology, including photodynamic therapy, chemodynamic therapy and photothermal therapy have attracted much attention [3, 8, 9]. Thereby, researchers have proposed myriads of novel nanoplatforms with multitudinous compositions and structures to continuously provide new modalities for cancer treatment [10–13]. Among multiple nanotherapeutic approaches, chemodynamic therapy (CDT) which can *in-situ* transform endogenous reactive substrate (H_2_O_2_) into toxic tumor-killing matters (·OH), has attracted extensive research interest for its minimized toxicity to non-lesion sites, reduced drug resistance and robust lethality [6, 7, 14–24]. However, tumor microenvironment (TME) in which tumor cells exist features not only acidity [25, 26] and overexpression of H_2_O_2_ [27], but also overexpression of GSH (up to 10 × 10^− 3^ M) [28] for regulating redox equilibrium in the TME. The robust anti-oxidation property endowed by high concentration of GSH severely impedes the effectiveness of CDT. Therefore, it is urgent to develop a novel therapeutic platform combined with GSH depletion to achieve desirable CDT efficiency.

Mesoporous silica (mSiO_2_)-based CDT nanoplatforms have attracted a great deal of interests for their potential in combining the high drug loading capacity endowed by mSiO_2_ and efficient CDT of functional nanomaterials with enzyme-like activity to achieve combined CDT [14, 23, 29–32]. Most of these composite nanoplatforms adopted core@shell structure, co-assembly or post-loading/grafting strategies. However, the aforementioned structures possess inherent shortage that different functional units in a nanoplatform cannot exert their functionalities to the maximum extent due to interference between them, thus severely retarding the effectiveness of combined therapy [33–37]. Consequently, it is necessary to construct a novel structure to achieve the combination of the advantage of mSiO_2_ and functional nanomaterials and isolation of their functions.

**Fig. 1:**
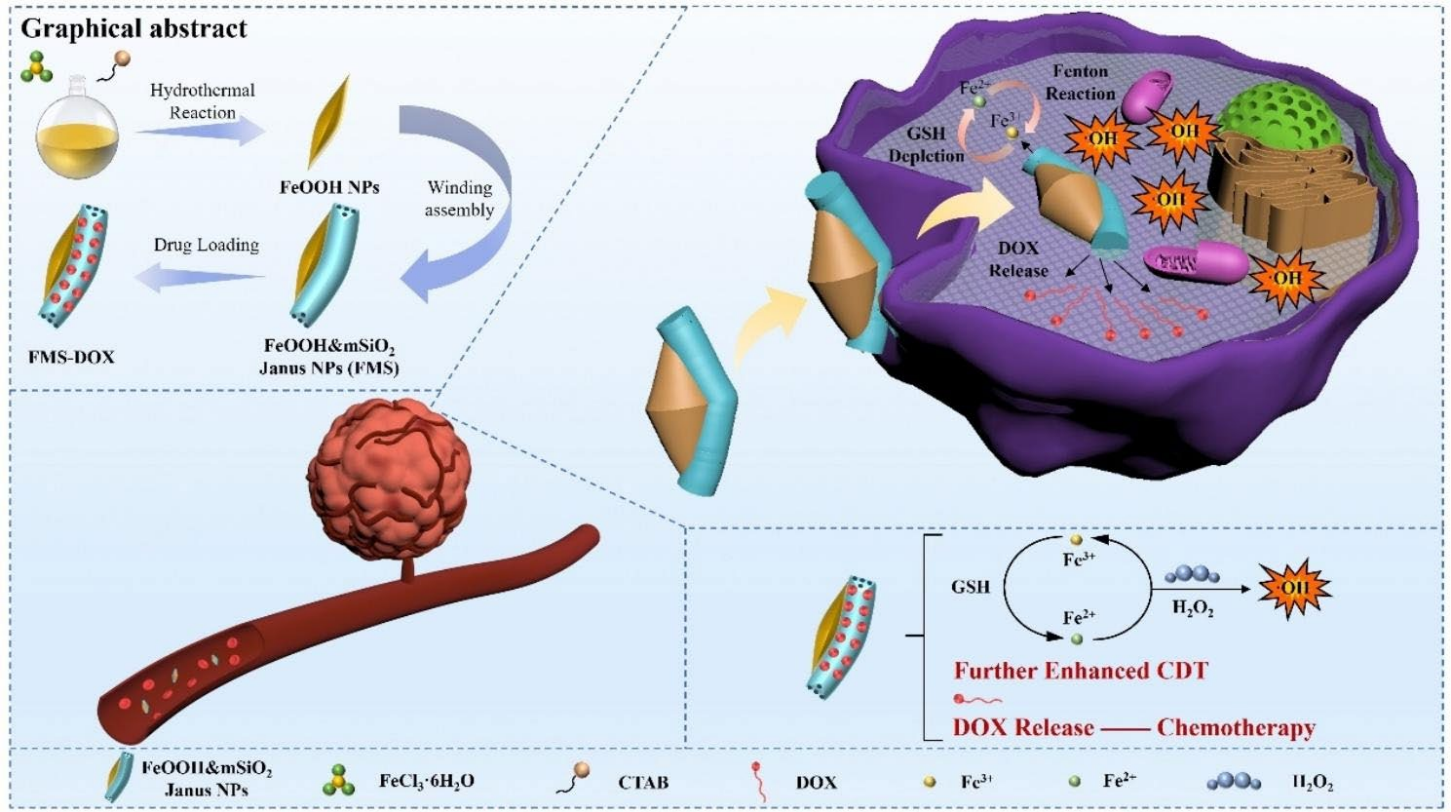
Schematic illustration of sushi-like Janus mesoporous nanoplatform for combination therapy of a tumor

Herein, inspired by the unique combination of rice and diverse ingredients in sushi, we developed a surface curvature-induced oriented assembly strategy to synthesize sushi-like mesoporous Janus composite nanomaterials. The nanoplatform was constructed by oriented assembly of mSiO_2_ nanorods on FeOOH nanospindles, followed by the loading of anticancer drug doxorubicin (DOX), forming pristine and DOX-loaded FeOOH&mSiO_2_ Janus composite nanoparticles (abbreviated as FMS and FMS-DOX, respectively). The synthesized FMS is structurally composed of two functional units, a spindle-shaped FeOOH CDT subunit with a length of about 400 nm and a diameter of about 50 nm and a rod-like mSiO_2_ drug-loading subunit with about 550 nm in length and 100 nm in diameter. The FeOOH CDT subunit is half covered by mSiO_2_ nanorod along its long axis, forming sushi-like nanostructure. The length and width of the mSiO_2_ nanorod can be tuned in a wide range to achieve a series of sushi-like structures. Such kind of intelligent therapeutic nanoplatform possesses enhanced CDT and drug delivery for efficient tumor inhibition by TME-activable sequential catalytic reactions (Fig. 1). Once FMS nanoparticles were endocytosed into cancer cells, the reductive GSH in TME can trigger the reduction of FeOOH, enabling the conversion of Fe^3+^ to Fe^2+^ which further catalyze overexpressed H_2_O_2_ in TME into highly lethal ·OH for efficient CDT. Meanwhile, GSH is depleted and combined with DOX release in acidic environment, which further exemplifies oxidative stress to tumor cells, thus achieving efficient tumor inhibition by combined therapy. Based on such a novel therapeutic nanoplatform, in vitro eliminating efficiency of tumor cells is as high as ~ 87%, in vivo experiments further show the efficient inhibition of tumor, verifying the synthesized FMS as a promising therapeutic nanoplatform.

## Results & discussion

### Synthesis and characterization of FMS

**Fig. 2:**
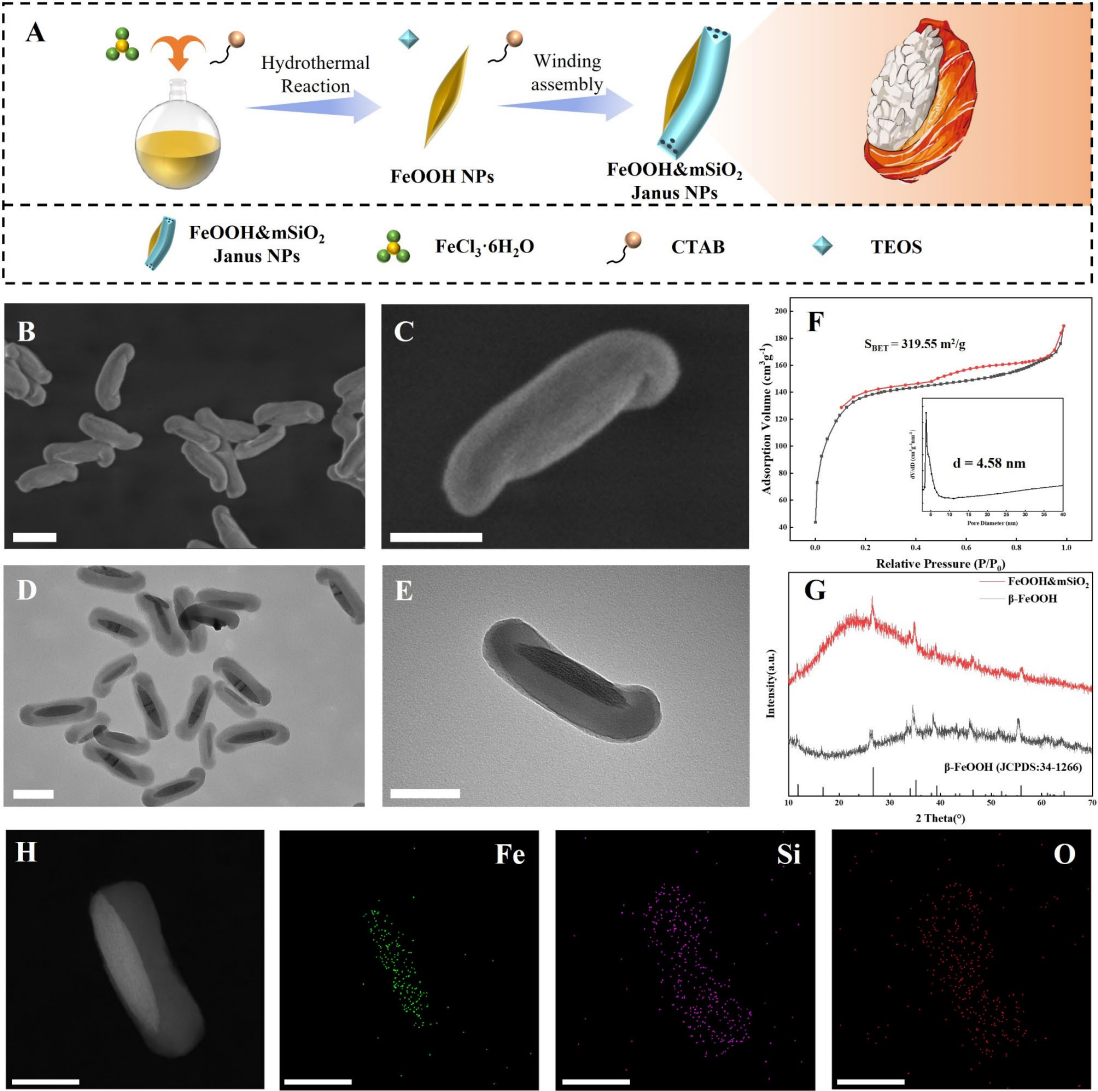
Characterizations of mesoporous FeOOH&mSiO_2_ Janus nanoparticles. (**A**) Schematic illustration of the synthetic process of FeOOH&mSiO_2_ Janus nanoparticles. (**B, C**) SEM and (**D, E**) TEM images with different magnifications of the obtained FeOOH&mSiO_2_ Janus nanoparticles. (**F**) Nitrogen sorption isotherms and the pore size distribution of FeOOH&mSiO_2_ Janus nanoparticles. (**G**) PXRD Patterns of pre-synthesized β-FeOOH and FeOOH&mSiO_2_ Janus nanoparticles. (**H**) Elemental mapping of Fe, Si and O in the FeOOH&mSiO_2_ Janus nanoparticles. Scale bar: 200 nm

FeOOH nanospindles with a diameter of ~ 400 nm in length and 50 nm in diameter were first fabricated through a previously reported hydrothermal method [38] (Supplementary Fig. 1). Then, mesoporous silica nanorods (mSiO_2_) are oriented grown along the long-axial curvature of the FeOOH nanospindles, forming sushi-like nanostructure. Scanning electron microscope (SEM) and transmission electron microscope (TEM) images depict that mSiO_2_ nanorod with a diameter of ~ 100 nm winds along the axial curvature of FeOOH nanospindle and extends slightly at the ends of the long axis of the nanospindle, covering half of it (Fig. 2B and E). The length of the mSiO_2_ nanorods is about 550 nm, large-area SEM image shows that the asymmetric structure was synthesized with good morphological purity and dispersity (Supplementary Fig. 2). Such a winding-made sushi-like structure confers a strong binding force to the two functional units, thus ensuring superior structural stability (Supplementary Fig. 3). Elemental mapping results clearly show the uneven distribution of Fe and Si in the nanoparticle, verifying the sushi-like Janus structure (Fig. 2H, Supplementary Fig. 4). Energy dispersive spectroscopy (EDS) result indicate that the Si/Fe atomic ratio of FeOOH&mSiO_2_ Janus nanoparticle is about 5:1 (Supplementary Fig. 5). The nitrogen sorption isotherm of the obtained FeOOH&mSiO_2_ Janus nanoparticles features a type-IV isotherm with a hysteresis loop at a relative pressure of 0.4–0.7, which is contributed by the ordered mesopores in the mSiO_2_ nanorod subunits (Fig. 2F). The Brunauer − Emmett − Teller (BET) surface area and Barrett − Joyner − Halenda (BJH) pore size of FeOOH&mSiO_2_ Janus nanoparticles are calculated to be 319.55 m^2^/g and ∼4.6 nm, respectively. High magnification TEM image shows that the mesoporous channels parallel to the long-axial direction of FeOOH (Supplementary Fig. 6). Powder X-ray diffraction (PXRD) patterns show no difference between the peaks of pristine FeOOH and FeOOH&mSiO_2_ Janus nanoparticles (Fig. 2G), both of them are consistent with tetragonal β-FeOOH phase (JCPDS 34-1266).

**Fig. 3:**
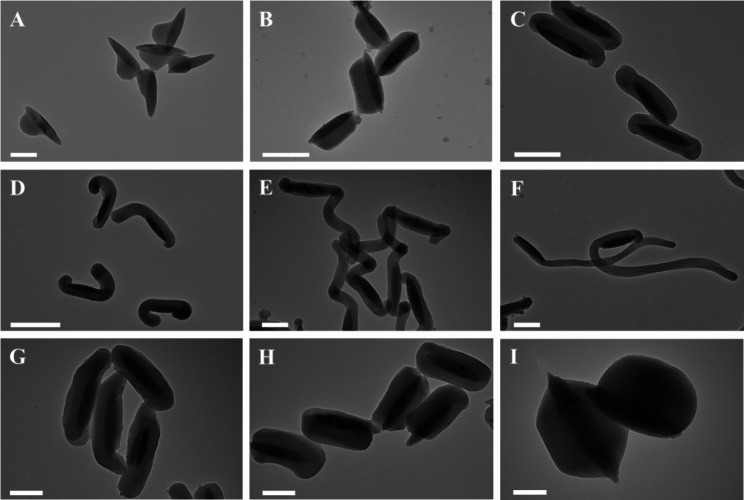
TEM images of FeOOH&mSiO_2_ Janus nanoparticles synthesized in different reaction conditions. (**A**) 2.7 mM CTAB; (**B**) 6.9 mM CTAB; (**C**) 9.6 mM CTAB; (**D**) 13.7 mM CTAB; (**E**) 20.6 mM CTAB; (**F**) 27.4 mM CTAB; (**G**) 3% (v/v) NH_3_·H_2_O; (**H**) 4% (v/v) NH_3_·H_2_O; (**I**) 8% (v/v) NH_3_·H_2_O. All conditions are fixed as experiment section in Supporting Information except for the altered one. Scale bar: 200 nm

The synthetic method exhibits excellent tunability. A series of FeOOH&mSiO_2_ Janus nanoparticles with controllable morphologies can be synthesized by simply adjusting the additive amount of CTAB and NH_3_·H_2_O. As shown in Fig. 3A F, a series of FeOOH&mSiO_2_ Janus nanoparticles winded with different length of mSiO_2_ nanorods were synthesized by manipulating the concentration of CTAB in the reaction solution. The length of mSiO_2_ increased accordingly when the amount of CTAB is upregulated. The island-like mSiO_2_ subunit can be clearly observed when the concentration of CTAB is controlled at a relatively low level (1.4 mM − 2.7 mM), forming axe-like Janus nanocomposite with FeOOH (Fig. 3A, Supplementary Figs. 7–8). Sushi-like structure can be obtained when the concentration of CTAB is controlled at an appropriate level of 6.9–9.6 mM (Fig. 3B C, Supplementary Fig. 9). In contrast, when the concentration of CTAB is further increased to 10.8 mM or even higher (20.6 mM − 27.4 mM), mSiO_2_ nanorods covering on the FeOOH nanospindles grows thinner and longer. Long tails can be formed and disengage from the curvature of FeOOH nanospindles (Fig. 3D F, Supplementary Figs. 10–12). The diameter of the mSiO_2_ nanorods can also be finetuned by adjusting the concentration of NH_3_·H_2_O. Mesoporous silica nanorods cannot be formed when the amount of NH_3_·H_2_O is lower than 2% (Supplementary Figs. 13–14), only resulting in the uniform coating layers on the FeOOH nanospindles to form core@shell structure. The diameter of mSiO_2_ nanorods is about 200 nm when the amount of NH_3_·H_2_O is increased to 3%. When the amount of NH_3_·H_2_O is further increased to 4% or 8%, the diameter of mSiO_2_ nanorods was further increased to ~ 300 or 400 nm, respectively (Fig. 3G H, Supplementary Figs. 15–17). An excessive amount of NH_3_·H_2_O (10-20%, Supplementary Figs. 18–19) leads to phase separation. Synthetic temperature and the amount of TEOS also have a significant influence on the morphology of synthesized nanocomposite (Supplementary Figs. 20–26). Such a self-assembly strategy can also be applied to other nanoparticles with rod-like structure, such as Bi_2_S_3_, Au nanorod and so on, sushi-like structure can be obtained in similar reaction conditions, demonstrating the good versatility of this strategy (Supplementary Figs. 27–28).

**Fig. 4:**
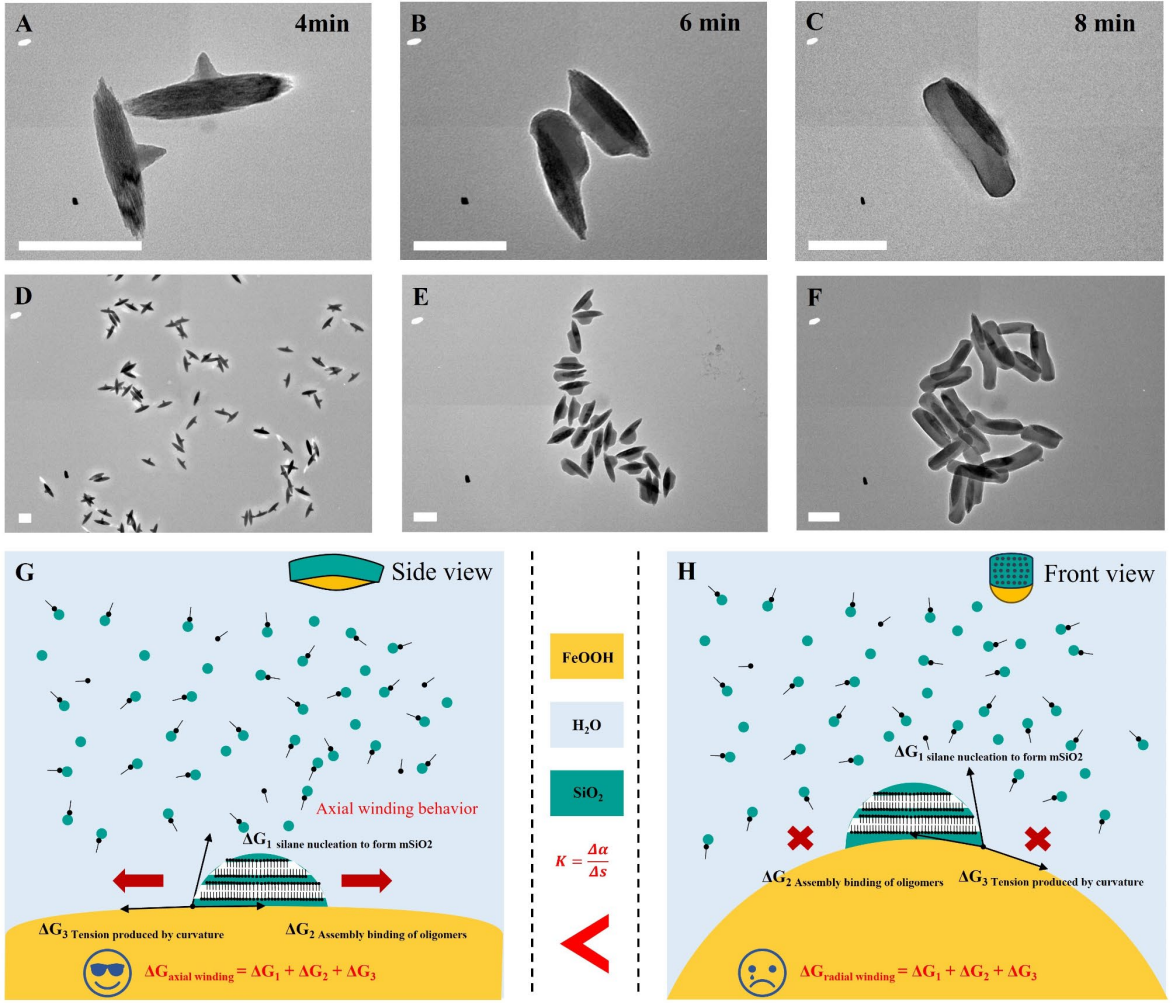
Mechanism of surface curvature-induced oriented assembly. TEM images of products obtained at different reaction stages: fixed-sampling of reaction process. (**A, D**) 4 min. (**B, E**) 6 min. (**C, F**) 8 min. (**G**) Mechanistic analysis of radially winding growth behavior from the side view. (**H**) Mechanistic analysis of transversal growth from the front view. Scale bar: 200 nm

All the data reveals that mSiO_2_ nanorod can “recognize” the long axis of the FeOOH nanospindle and always grows axially into sushi-like structure. In contrast to conventional mesoporous Janus nanoparticles, such kind of anisotropic growth is site-selective. As shown in Fig. 4A, at the initial stage of the reaction, mSiO_2_ first nucleates near the center of the long axis of the nanospindle, forming a “T”-shaped structure, and the directionality of the mesoporous silicon growth is not visible at this point. The mSiO_2_ nanorods then grow along the long axis of the FeOOH nanospindle, although most of the rods grow toward one end first, forming a deviated structure (Fig. 4E). Eventually, the silica nanorods cover the long axis of the FeOOH nanospindle completely, forming a sushi-like structure. It can be seen that mSiO_2_ nanorods spontaneously undergo a direction-selective growth, rather than being concentrated on the long axis by rearrangement. Furthermore, it was demonstrated that the growth of other forms of silica with disordered mesotructure (nonporous, radial porous) exhibit no similar oriented-growth selectivity (Supplementary Figs. 29–30). On the contrary, the growth of periodic mesoporous organosilicon (PMO) with cubic mesostructure exhibits similar long-axis selectivity as mSiO_2_ nanorod (Supplementary Fig. 31), thus we speculate that the ordered mesostructure of the mSiO_2_ nanorod plays a key role in oriented-growth selectivity.

Based on the above experimental results, we propose the surface curvature-induced oriented assembly mechanism from the perspective of reaction thermodynamics, so as to explain the long-axis selective growth phenomenon in the sushi-like structure. The free energy change (ΔG) during the whole mesoporous silica nanorod growth process can be divided into three parts: (1) ΔG_1_ for the energy change during silane’s nucleation to form mesoporous silica. (2) The formation of a new interface between silica and FeOOH, there is an interfacial energy change ΔG_2_. (3) The energy required for the ordered mesostructure to curve along the interface, ΔG_3_.

Formulated as: ΔG = ΔG_1_ + ΔG_2_ + ΔG_3_. The system tends to evolve in a ΔG-minimal way.

Among the three energy changes, ΔG_1_ depends mainly on synthetic conditions but not FeOOH nanospindle, so G_1_ is the same between long and short axis directions. While ΔG_2_ and ΔG_3_ are both related to the interface and influenced by the surface curvature of precursor. However, ΔG_2_ interfacial energy is an atomic-level interaction and is less affected by nanoscale interfaces, thus G_2_ does not change much with growing direction. The main factor affecting ΔG_3_ is the assembly of CTAB micelles on the solid surface. The CTAB micelles can be regarded as nanorods with a diameter of about 3 nm, and the surface curvature of the FeOOH nanospindle is of the same order of magnitude, so ΔG_3_ is significantly influenced by the curvature. As can be seen in Fig. 4G H, the surface curvature along the long axis direction (side view) is small and can be nearly regarded as flat, while the curvature in the short axis (front view) direction is way larger, so ΔG_3short_ > ΔG_3long_. The total ΔG_long_ in the long axis direction is smaller than ΔG_3short_ in the short axis direction, so the mesoporous silica nanorods are assembled and grown along the long axis and stop at both ends to obtain a sushi-like structure, from which we can reasonably explain afore-mentioned experimental results.

This mechanism explains the previously observed experimental phenomenon that the oriented growth of mSiO_2_ is always present simultaneously with ordered mesostructure. The ΔG required for bending the ordered mesostructure on the surface of FeOOH nanospindles is the main cause of directional selectivity. Therefore, regardless of changes in length or diameter of the mSiO_2_ nanorods or even cubic mesostructure, the mSiO_2_ always undergo oriented growth along the long axis direction of the FeOOH nanospindles (i.e. the surface with minimal curvature). Once the silica no longer grows with ordered mesostructure (such as the radially orientated porous or nonporous structure), the ΔG does not involve the variable related to the curvature of the mesostructure (ΔG_3_). Thus, the ΔG of the mesoporous silica growing at different positions on the FeOOH nanospindle is nearly the same, the oriented growth characteristic disappears.

### Catalytic and drug-loading properties of FMS

**Fig. 5:**
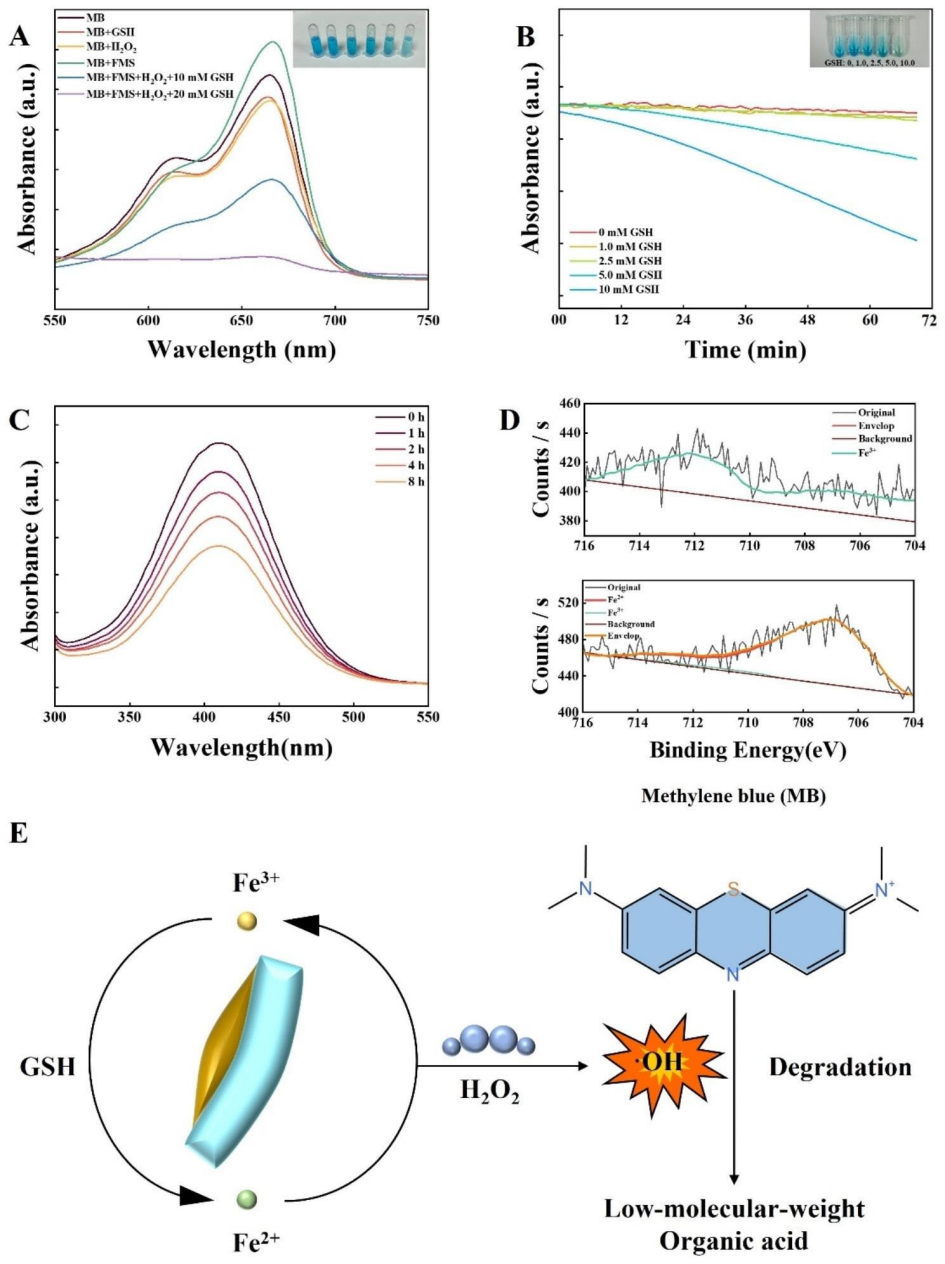
Catalytic and drug-loading properties of FeOOH&mSiO_2_ Janus nanoparticles. (**A**) Absorption curve of methylene blue (MB) under different catalytic conditions (10 mM GSH, 10 mM H_2_O_2_, 125 µg/mL FMS, 125 µg/mL FMS + 10 mM H_2_O_2_ and 125 µg/mL FMS + 10 mM H_2_O_2_ + 10 mM GSH, respectively) determined by UV-vis absorption spectroscopy after being co-incubated for 3 h. The inset picture is the optical photograph of experimental group placed in absorbance-descending order. (**B**) Time-dependent curve of MB degradation under different GSH concentrations (0, 1.0, 2.5, 5.0, 10.0 mM). The inset picture is the optical photograph of experimental group placed in absorbance-descending order. (**C**) GSH (1 mM) depletion by FeOOH&mSiO_2_ Janus nanoparticles determined by UV-vis absorption spectroscopy with DTNB as a probe. (**D**) X-ray photoelectron spectroscopy analysis of FMS and GSH-treated FMS (upper the former, lower the latter). Fe^2+^ and Fe^3+^ refer to standard fit peaks. Original XPS signals are weak because of the low content of Fe element in FMS samples (~ 10%). GSH concentration: 10 mM. (**E**) Illustration of the Fe^3+^-Fe^2+^ reaction circulation, in which Fe^3+^ depletes GSH, Fe^2+^ catalyzes Fenton reaction and degrades MB. All conditions are fixed as the experiment section except for the altered one. MB concentration: 10 µg/mL. pH: 5.4

To further investigate the catalytic properties of FeOOH&mSiO_2_ Janus nanoparticles, methylene blue (MB) was used as a probe to assess the generation of hydroxyl radical (·OH) under the catalysis of FMS. In a weak acidic environment (pH 5.4), the FeOOH subunits in FMS can catalyze H_2_O_2_ to generate ·OH, which is demonstrated by the degradation of MB, symbolled by the visibly diminished blue color of the reaction solution and significantly weakened absorption peak at 665 nm from UV-vis absorption spectrum (Fig. 5A and B). MB was not degraded by FMS or H_2_O_2_ alone, and was partially degraded with the presence of both FMS and H_2_O_2_. MB degradation reaches the maximum extent under the catalysis of FMS in the presence of both H_2_O_2_ and GSH. It can be seen in the inset optical photograph in Fig. 5A that the blue color of MB solutions varied significantly under different catalytic condition. Under the catalysis of FMS, MB is completely degraded in 6 h, and the degradation of MB gradually expedites with time (Supplementary Fig. 32A). The catalytic performance of FMS exhibits a positive correlation to the concentration of H_2_O_2_ (Supplementary Fig. 32B). As shown in Supplementary Fig. 32C, the velocity of MB degradation is also related to the concentration of GSH, which gradually increases accordingly when the concentration of GSH is upregulated. Thus, we can conclude that GSH can enhance the catalytic properties of Janus nanocomposites.

We then further investigated the role of GSH in the enhanced catalytic performance. The depletion of GSH was investigated by utilizing 5,5′-dithiobis-(2-nitrobenzoic acid) (DTNB) as a molecular indicator which could be reduced by GSH to generate yellow 2-nitro-5-thiobenzoic acid (TNB) with a characteristic absorbance at 412 nm [39] (Fig. 5C). In the presence of FMS nanoparticles, the GSH content gradually decreased with the extension of incubation time, and about ~ 45% of the GSH in the solution was consumed in 8 h. X-ray Photoelectron Spectroscopy (XPS) analysis also shows that Fe^3+^ of FMS is almost completely conversed to Fe^2+^ after co-incubating with GSH for 6 h, which further proved the GSH depletion effect of FeOOH functional units in FMS (Fig. 5D). It has been demonstrated that Fe^2+^ ion has a higher Fenton activity than Fe^3+^ ion [40–45]. So we can conclude that Fe^3+^ consumes GSH while being converted to more active Fe^2+^, and the as-generated Fe^2+^ is further oxidized to Fe^3+^ when catalyzing the generation of ·OH, and this cycle goes on to facilitate a GSH depletion & CDT combined therapy (Fig. 5E).

With abundant mesopores, mSiO_2_-based FMS can be utilized to load therapeutic agents to achieve combined chemotherapy. As one of the most prevailing chemotherapy drugs, Doxorubicin (DOX) is loaded into FeOOH&mSiO_2_ Janus nanoparticles (FMS-DOX). The success loading of DOX can be proved by fluorescence spectra of FMS, DOX and FMS-DOX (Supplementary Fig. 33A). The loading capacity of DOX were measured by UV/vis spectrum and calculated to be ~ 40 wt% (at DOX/FMS ratio of 2:1), and it drastically decreases from ~ 80 wt% to ~ 40 wt% according to the adjustment of feeding ratio of DOX/FMS (w/w) in an ascending order (Supplementary Fig. 33B). Besides, the DOX release is measured by UV/vis spectra. It can be seen that the DOX release is intensely dependent on the pH of the medium. At a pH of 5.4, DOX release is very fast in the first 2 h, reaching 60%. Then, the release slows down and gradually reaches above 80% in 24 h. In contrast, DOX only release ~ 30% in 24 h at pH of 7.2 (Supplementary Fig. 33C). In this way, the release of DOX can be synergized with the acid-catalyzed Fenton reaction to achieve combined chemotherapy-CDT treatment.

### In vitro therapeutic effect of FMS and FMS-DOX

**Fig. 6:**
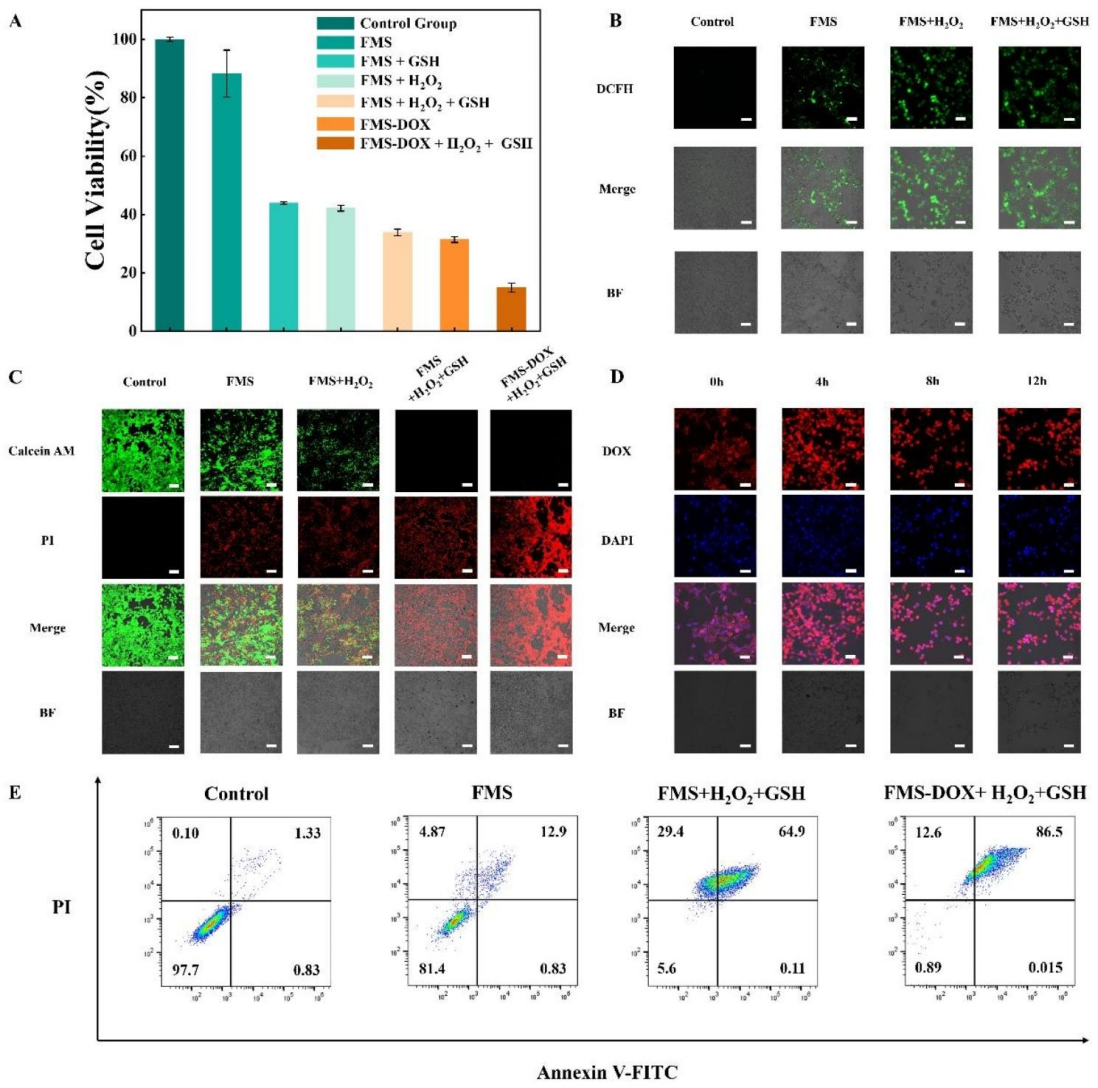
In vitro therapeutic effect of FeOOH&mSiO_2_ Janus nanoparticles conducted with 4T1 breast cancer cells. (**A**) Cell viability of 4T1 cells incubated with FMS/FMS-DOX/C-FMS under different conditions. (**B**) Confocal laser scanning microscopy (CLSM) images of DCFH-DA stained 4T1 cells after incubation with fresh medium and FMS with or without H_2_O_2_ and GSH after 8 h. (**C**) CLSM images of Calcein AM (green, live cells) and PI (red, dead cells) co-stained 4T1 cells after incubation with fresh medium, FMS/FMS-DOX with or without H_2_O_2_ and GSH for 12 h. (**D**) CLSM images of 4T1 cells stained with DAPI after incubation with FMS-DOX at different time points. (**E**) Flow cytometric quantitative analysis of Annexin V-FITC/PI co-stained 4T1 cells after co-incubation with FMS/FMS-DOX with or without H_2_O_2_ and GSH under weak acidic (pH 5.4) conditions for 12 h. The dosage of FMS and FMS-DOX in the in vitro experiments was all controlled at 200 µg/mL. The concentration of H_2_O_2_ and GSH in the in vitro experiments was all controlled at 10 mM. Scale bar: 100 μm

We then investigated the therapeutic effect of Janus nanoparticles on tumor cells. The in vitro cytotoxicity of FMS and FMS-DOX was performed on 4T1 cells by Cell Counting Kit-8 (CCK-8). Pristine FMS Janus nanoparticles have excellent biocompatibility and are not toxic to cells even at a high concentration of 200 µg/mL (Supplementary Fig. 34A). As shown in Fig. 6A and Supplementary Fig. 34B, the introduction of H_2_O_2_ and GSH to FMS can significantly decrease the cell viability, which can be attributed to the synergistic effect of GSH depletion and CDT. On the contrary, the co-incubation of 4T1 cells with pristine H_2_O_2_ or GSH didn’t result in significant cell apoptosis, proving the addition of exogenous H_2_O_2_ or GSH has minor effect on cells’ living (Supplementary Fig. 34C). The ability of eliminating tumor cells of FMS can be further enhanced by loading anti-cancer model drug doxorubicin (DOX), achieving ~ 91% eliminating efficiency of tumor cells (Supplementary Fig. 34D). Same tendency can also be observed on core@shell structured FeOOH@mSiO_2_ nanoparticles (C-FMS) which has weaker tumor cell elimination capability compared to sushi-like Janus FeOOH&mSiO_2_. The differences in tumor cell elimination efficiency can be attributed to the less-exposed active sites of FeOOH functional units and poorer mass transfer in the core@shell structure [14, 46] (Supplementary Fig. 35). The in vitro CDT effect of FMS was further demonstrated by a ROS probe of 2’,7’-dichlorodihydro-fluorescein diacetate (DCFH-DA), which can be deacetylated by intracellular esterase to nonfluorescent one, and then oxidized by ROS into green fluorescent 2’,7’-dichlorofuorescin (DCF) [23]. As can be seen in Fig. 6B, no obvious fluorescence was observed in the cells of control group, while the cells treated with FMS show faintish green fluorescence, the cells treated with FMS, H_2_O_2_ and GSH display bright and strong green fluorescence, demonstrating a robust intracellular ·OH production.

The in vitro combined therapeutic efficiency of FMS and FMS-DOX was also evaluated by incubating 4T1 cells with different samples for 12 h and then co-stained with Calcein acetoxymethyl ester (Calcein-AM) and propidium iodide (PI), which show green fluorescence for live cells and red fluorescence for dead cells respectively. As shown in Fig. 6C, the cells without any treatment (control group) grow very well, while a small portion of dead cells is observed from the cells incubated with FMS with or without H_2_O_2_, indicating the oxidative stress produced by the Fe^3+^-mediated Fenton reaction. More importantly, a large number of dead cells are observed from the group incubated with FMS, H_2_O_2_ and GSH, indicating the exemplified oxidative stress produced by Fe^2+^-mediated Fenton reaction, which can be attributed to the reduction of Fe^3+^ to Fe^2+^ accompanied by GSH depletion. Only red fluorescence (signals of dead cells) can be observed from the group incubated with FMS-DOX, H_2_O_2_ and GSH, indicating the superior therapeutic efficiency of the nanoplatform. The confocal laser scanning microscopic (CLSM) images show that the nanoparticles can carry and release DOX into the cell (Fig. 6D). After 4 h of co-incubation, the red fluorescence of DOX can be clearly observed around the nucleus (labeled with blue fluorescence), implying that DOX was carried by FMS and taken up by the cells together. At 12 h, the red and blue fluorescence almost overlapped, implying that DOX was released from FMS and entered into the nucleus. The annexin V-FITC/PI apoptosis detection kit was introduced to quantitatively analyze the cell apoptosis assay after different treatments. Obviously, the apoptosis rate of tumor cells in the FMS-DOX group with H_2_O_2_ and GSH could reach as high as ~ 99% after 12 h of incubation, which is much higher than that of the other groups (Fig. 6E), further demonstrating the excellent therapeutic efficacy of the nanoplatform. Same tendency can also be seen when the incubation time was further adjusted to 6 h (Supplementary Fig. 36). In comparison, core@shell structured FeOOH@mSiO_2_ (C-FMS) has lower therapeutic efficiency than asymmetric FMS, which can be observed on the flow cytometric quantitative analysis results of 4T1 cells co-incubated with C-FMS in similar chemical conditions (Supplementary Fig. 37). In addition, the intracellular GSH consumption has also been monitored quantitively with 5,5′-dithiobis-(2-nitrobenzoic acid) (DTNB). The results showed that intracellular GSH was consumed ~ 60% after 8 h of co-incubation with 200 µg/mL of FMS, proving the excellent GSH depletion efficiency of FMS nanoparticles (Supplementary Fig. 38).

In vivo **therapeutic effect of FMS and FMS-DOX**.

**Fig. 7:**
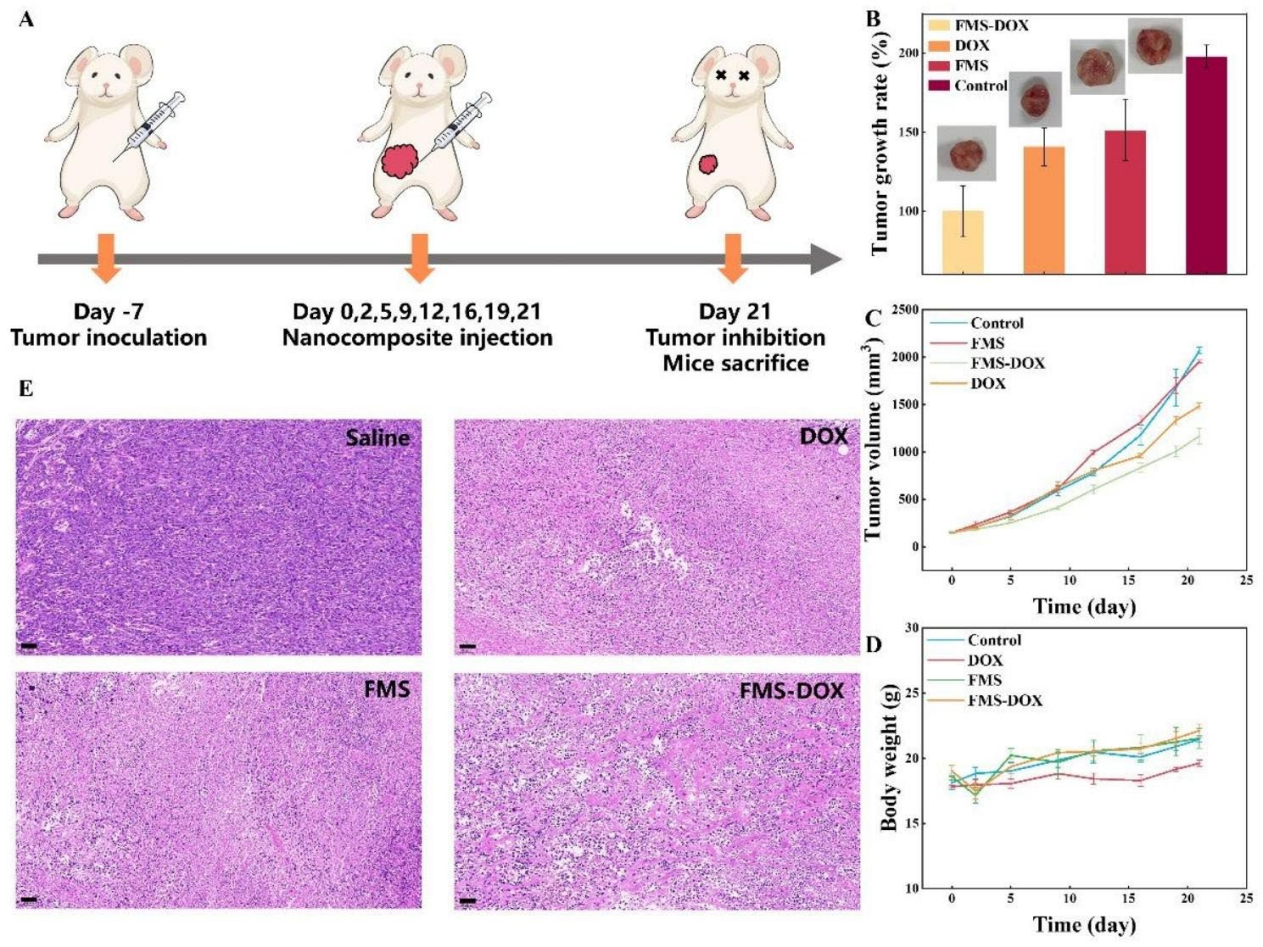
In vivo therapeutic efficacy of FeOOH&mSiO_2_ Janus nanoparticles conducted with 4T1-tumor-bearing mice. (**A**) Schematic illustration of tumor model establishment and treatment process. The nanocomposite was administered intravenously at 0, 2nd, 5th, 9th, 12th, 16th, 19th and 21st days. (**B**) Eventual tumor growth rate of 4T1 tumor-bearing mice treated with saline (control group), FMS, DOX and FMS-DOX calculated with dissected tumor weight (Normalized with the group treated with FMS-DOX). Data are expressed as mean standard ± errors (n = 3). (**C**) Tumor volume growth curves of 4T1 tumor-bearing mice treated with saline (control group), FMS, DOX and FMS-DOX. Data are expressed as mean standard ± errors (n = 3). (**D**) Body weights of tumor-bearing mice during treatments. Data are expressed as mean standard ± errors (n = 3). (**E**) H&E staining of tumor tissues harvested from corresponding mice after 21 days of treatments. Scale bar: 50 μm

Encouraged by the excellence of FMS-DOX in vitro therapeutic results, we then evaluated the cooperative therapeutic effect of FMS-DOX on 4T1-tumor-bearing mice. Hemolysis analysis was firstly conducted to evaluate the hemo-compatibility of FMS, it can be seen that all samples show negligible hemolytic effect even exposed to a high concentration of FMS (400 µg/mL), indicating the superior hemo-compatibility of prepared nanoparticles (Supplementary Fig. 39). As shown in Fig. 7A, the tumor-bearing mice were randomly divided into four groups for different treatments: saline (control group), DOX, FMS and FMS-DOX. Compared with the control group, the tumor growth is inhibited effectively after treating with FMS or FMS-DOX nanocomposite (Fig. 7B C). The group treated with FMS-DOX exhibits the most robust inhibition to tumor growth (~ 50%) due to combined CDT and chemotherapy compared with other groups (FMS: ~25%, DOX: ~30%), which can also be confirmed by the H&E staining assay and optical photographs (Supplementary Fig. 40). The body weight of treated mice exhibits no abnormal fluctuations, and no remarkable tissue damage or any other side effect is observed on heart, liver and kidney (Fig. 7E and Supplementary Fig. 41), demonstrating the excellent biocompatibility of the FeOOH&mSiO_2_ Janus nanoparticles, which is attributed to starkly different catalytic activities of the nanocomposite in normal physiological environment and tumor microenvironment.

## Conclusion

In summary, we have developed a surface curvature-induced oriented assembly strategy to synthesize a sushi-like mesoporous Janus composite materials composed of two subunits of a spindle-shaped FeOOH CDT subunit (~ 400 nm in length and ~ 50 nm in diameter) and a rod-shaped mSiO_2_ drug-loading subunit (~ 550 nm in length and ~ 100 nm in diameter) which covers half of the FeOOH nanospindle along its long axis. We elucidated how surface curvature affect the assembly of ordered mesostructure which leads to axis-selective growth of mSiO_2_ and synthesized a series of sushi-like Janus nanoparticles with varied morphologies. In acidic and reductive TME, Fe^3+^ of FMS-DOX is firstly reduced to Fe^2+^ by endogenous GSH, the as-generated Fe^2+^ then effectively catalyzes overexpressed H_2_O_2_ in TME into highly lethal ·OH, and DOX is then released to achieve combined CDT and chemotherapy. Meanwhile, the redox reaction between Fe^3+^ and endogenous GSH in TME can effectively recede the antioxidant capability of the tumor, further exemplifying the oxidative stress produced by ·OH to tumor cells. The combination of Fe^3+^-related GSH depletion, Fe^2+^-mediated ·OH generation, and DOX-induced chemotherapy endows FMS-DOX with highly efficient tumor inhibition capabilities in both *vitro and vivo*, providing a promising intelligent therapeutic nanoplatform for oncology. We believe that the surface curvature-induced oriented assembly strategy proposed here can also be an intriguing and enlightening strategy for the construction of more novel nanoplatforms with unique isolated functional units.

## Materials and methods

### Chemicals and materials

Iron (III) chloride hexahydrate (FeCl_3_·6H_2_O, AR), phosphate buffered saline (PBS), methylene blue (MB), tetraethyl orthosilicate (TEOS, AR), hexadecyl trimethyl ammonium Bromide (CTAB, 99%), and 5,5′-dithiobis-(2-nitrobenzoic acid) (DTNB) were all purchased from Aladdin. Ammonium Hydroxide (NH_3_·H_2_O) and ethanol were purchased from Sinopharm Chemical Reagent Co., Ltd. Cell Counting Kit-8 (CCK-8), Calcein-AM/propidium iodide (PI), 4’,6-diamidino-2-phenylindole (DAPI), 2,7-Dichlorodihydrofluorescein diacetate (DCFH-DA), Annexin V-FITC Apoptosis Detection Kit were obtained from Beyotime Biotech (China). Penicillin-Streptomycin and Dulbecco’s modified Eagle medium (DMEM) were purchased from HyClone (USA). Fetal bovine serum was purchased from Gibco (USA). All chemicals in this work were used as received without further purification. Deionized water (18.2 MΩ·cm, 25 °C) was used in all experiments, and all solutions were freshly prepared for immediate use in each experiment.

### Synthesis of FeOOH nanospindles

FeOOH nanospindles were synthesized by a hydrothermal method according to previous research [38]. Briefly, 2.7 g FeCl_3_·6H_2_O and 2.5 g CTAB were dispersed in 100 mL H_2_O. The mixture was then transferred into a 250 mL glass flask. The flask was transferred into an oven, heated to 60 °C and maintained for 12 h. After cooling down to room temperature, the product was centrifuged, washed with distilled water and ethanol, and finally dispersed in ethanol for further usages.

### Synthesis of FeOOH&mSiO_2_ Janus nanoparticles

For the fabrication of FeOOH&mSiO_2_ Janus nanoparticles with sushi-like structure, 8 mg of the obtained FeOOH nanoparticles were dispersed in 10 mL of deionized water followed by the addition of 9.6 mM (35 mg) CTAB. After ultrasonication for 30 min, the solution was transferred to 313 K oil bath under constant magnetic stirring of 500 rpm. NH_3_·H_2_O (5% (v/v)) was then added to the solution, followed by the dropwise addition of 80 µL TEOS. The above solution was allowed to react for 1 h. The FeOOH&mSiO_2_ Janus nanoparticles were centrifuged and washed by ethanol for 3 times to remove excessive CTAB. Subsequently, the products were redispersed in 25 mL of ethanol for further use.

### Fenton reaction catalytic activity experiments

The Fenton reaction catalytic activity of FeOOH&mSiO_2_ Janus nanoparticles were detected using methylene blue (MB) as the probe in the presence of H_2_O_2_ and GSH. FeOOH&mSiO_2_ Janus nanoparticles (125 µg/mL), GSH (0, 1, 2.5, 5, 10 mM), H_2_O_2_ (10 mM) and MB (10 µg/mL) were added into 1 mL of PBS (pH 5.4). The absorption peak of MB at 665 nm was recorded by a microplate reader.

### Cell culture

Murine breast cancer cell line (4T1 cells) was purchased from cell bank of Chinese academy of science (Shanghai, China). The cells were cultured in standard Penicillin-Streptomycin and Dulbecco’s modified Eagle medium (DMEM) supplemented with 10% (v/v) FBS, 100 µg/mL streptomycin and 100 U/mL penicillin at 37 °C in a humidified incubator with 5% CO_2_.

### In-vitro cell uptake of DOX-loaded FeOOH&mSiO_2_

The intracellular endocytosis of FeOOH&mSiO_2_ Janus nanocomposites was investigated by confocal laser scanning microscopy (CLSM). For CLSM observation, 4T1 cells were seeded in the CLSM-exclusive culture dishes (105 cells per dish) and incubated for 24 h. Then, DOX-loaded FeOOH&mSiO_2_ nanocomposites (200 µg/mL) were added into the culture media. After co-incubation for 0, 4, 8, 12 h, the cells were washed with PBS. Then, the cells were stained with 4’,6-diamidino-2-phenylindole (DAPI) for 10 min and imaged by CLSM. Moreover, the cells were collected by trypsin digestion and transferred into the test tube for the quantitative analysis of the fluorescence signal by flow cytometry.

### In-vitro cytotoxicity evaluation

The cytotoxicity of the prepared FeOOH&mSiO_2_ nanocomposites was assessed using standard Cell Counting Kit-8 (CCK-8) assay. 4T1 cells were seeded in 96-well plates (105 cells/well) for 24 h. After that, the cells were incubated with fresh medium containing different concentrations of FeOOH&mSiO_2_ nanocomposites (200, 100, 50, 25, 12.5, 6.25, 3.125 µg/mL) with or without H_2_O_2_ (10 mM) and GSH (10 mM) for another 12 h. Then, the medium was discarded and the cells were washed with PBS. The mixture of CCK-8 and fresh culture medium was added into each well and incubated for 2 h. Finally, the cell viability was evaluated by measuring the absorbance at the wavelength of 450 nm.

### In-vitro therapeutic efficiency

Confocal laser scanning microscopic (CLSM) and flow cytometry were introduced to evaluate the in-vitro therapeutic efficacy of FeOOH&mSiO_2_ and DOX-loaded FeOOH&mSiO_2_ Janus nanocomposites. 4T1 cells were seeded in the CLSM-exclusive culture dishes (105 cells per dish) and incubated for 24 h. The cells were then co-incubated with FeOOH&mSiO_2_ or DOX-loaded nanocomposites (200 µg/mL) with or without H_2_O_2_ (10 mM) and GSH (10 mM) in the DMEM culture medium (pH 5.4). After 24 h, the cells were washed with PBS for three times, stained with Calcein-AM/PI after different treatments for CLSM observation. The quantitative analysis of the Cell apoptosis was determined by flow cytometry after co-incubation with Annexin V-FITC/PI apoptosis detection kit in dark for 20 min.

### Hemolysis analysis

4 mL of fresh anticoagulation mice blood sample was collected and diluted with 5 mL PBS solution. 1 mL of nanocomposite suspension (12.5, 25, 50, 100, 200, 400, 800 µg/mL) was incubated in the 37 ℃ water bath for 30 min, and then 1 mL of diluted blood was added to the nanocomposite suspensions, following by shaking for 180 min sin the water bath, and then samples were centrifuged (4500 r/min, 4 min). The absorbance of supernatants at 541 nm were measured by a microplate reader. Red blood cells treated with ultrapure water and PBS solution were set as negative and positive controls.

### Tumor model

All the animal experiments were approved by the Shanghai Science and Technology Committee and performed in agreement with the guidelines of the Department of Laboratory Animal Science, Fudan University. 4 ~ 6 weeks-old Female Balb/c mice were commercially supplied by Shanghai JSJ Laboratory Animal Co. Ltd. (Shanghai, China). 4T1 cells suspended in FBS (1 × 107 cells) were subcutaneously injected into the right back leg of mice. When the tumor volume has grown to ~ 100 mm^3^, the tumor-bearing mice were randomly divided into 4 groups (n = 3 for each group): control, FMS, DOX and FMS-DOX. Saline and its solution of FMS, DOX and FMS-DOX were intravenously injected to four groups of mice respectively at 0, 2nd, 5th, 9th, 12th, 16th, 19th and 21st day, and the body weights and tumor sizes of each group were monitored periodically. All mice were sacrificed and dissected at 21st day, organ sections were stained with Hematoxylin and eosin (H&E) for observation.

### Apparatus and characterizations

Transmission electron microscopy (TEM) observations were acquired on JEM-2100 F with an accelerating voltage of 200 kV equipped with a post-column Gatan imaging filter (GIF-Tridium). Scanning electron microscopy (SEM) images were taken using a Hitachi S-4800 ultrahigh resolution cold FEG with an in-lens electron optic operating at 20 kV. Nitrogen adsorption-desorption measurements were conducted to obtain information on the porosity. The measurements were conducted at 77 K with ASAP 2420 analyzer (USA). The UV/Vis spectra were recorded on Lambda 35 Perkin-Elmer. Confocal fluorescence images were obtained by an LSM 980 confocal laser scanning microscope (Carl Zeiss SMT Inc., USA). Flow cytometry analysis was performed by an Accuri C6 flow cytometer (BD Biosciences, USA). The fluorescence spectra were measured on an Edinburgh FLS980 spectroscope, with all the nanoparticles dispersed in water to form a transparent colloidal dispersion.

### Electronic supplementary material

Below is the link to the electronic supplementary material.

Supplementary Material 1

## Data Availability

The data that support the findings of this study are available from the corresponding authors upon reasonable.

## Abbreviations

TME: Tumor microenvironment
NP(s): Nanoparticle(s)
CDT: Chemodynamic therapy
DIW: Deionized water
DOX: Doxorubicin
CTAB: hexadecyl trimethyl ammonium Bromide
TEOS: tetraethyl orthosilicate
DTNB: 5,5′-dithiobis-(2-nitrobenzoic acid)
MB: Methylene blue
GSH: Glutathione
mSiO_2_: mesoporous silica
FMS(s): FeOOH&mSiO_2_ Janus nanoparticle(s)
FMS-DOX: DOX-loaded FeOOH&mSiO_2_ Janus nanoparticle
C-FMS(s): Core@shell structured FeOOH@mSiO_2_ nanoparticle(s)
PBS: Phosphate buffered saline
CCK-8: Cell counting kit 8
Calcein AM/PI: Calcein acetoxymethyl ester/propidium iodide
DAPI: 4’,6-diamidino-2-phenylindole
DCFH-DA: 2,7-Dichlorodihydrofluorescein diacetate
DMEM: Dulbecco’s modifed Eagle’s medium
FBS: Fetal bovine serum
PXRD: Powder X-ray diffraction
SEM: Scanning electron microscopy
TEM: Transmission electron microscopy
EDS: Energy dispersive spectroscopy
BET: Brunauer − Emmett − Teller
BJH: Barrett − Joyner − Halenda
XPS: X-ray photoelectron spectroscopy
CLSM: Confocal laser scanning microscopy
FLS: Fluorescence spectrum
H&E: Hematoxylin and eosin
