# Supplementary material for: Surface curvature-induced oriented assembly of sushi-like Janus therapeutic nanoplatform for combined chemodynamic therapy
Source: J Nanobiotechnology. 2023 Nov 15;21:425. doi: 10.1186/s12951-023-02138-0 (PMC10647176; doi:10.1186/s12951-023-02138-0)
Supplement: Supplementary file 1 — Supplementary Material 1 [file 12951_2023_2138_MOESM1_ESM.docx]

**Supplementary Material**

**Surface Curvature-induced oriented assembly of** **sushi-like Janus therapeutic nanoplatform for combined chemodynamic therapy**

Yanming Ma ^1^, Minchao Liu ^1^, Mengmeng Hou ^1^, Yufang Kou ^1^, Wenxing Wang ^1*^, Tiancong Zhao ^1*^, and Xiaomin Li ^1*^

^1^ Department of Chemistry, Laboratory of Advanced Materials, College of Chemistry and Materials, Shanghai Key Laboratory of Molecular Catalysis and Innovative Materials, State Key Laboratory of Molecular Engineering of Polymers, Collaborative Innovation Center of Chemistry for Energy Materials (2011-iChEM), Fudan University, Shanghai 200433, China.

Corresponding authors: [wangwenxing@fudan.edu.cn](mailto:12113010002@fudan.edu.cn); [zhao_tc@fudan.edu.cn](mailto:zhao_tc@fudan.edu.cn); [lixm@fudan.edu.cn](mailto:lixm@fudan.edu.cn)

**Table of Contents for Supporting Material**

[Supplementary Methods S2](#_Toc135077128)

[Supplementary Figures S7](#_Toc135077129)

[Supplementary References S21](#_Toc135077130)

# Supplementary Methods

**Materials:**

Iron (III) chloride hexahydrate (FeCl_3_·6H_2_O, AR), tetraethyl orthosilicate (TEOS, AR), hexadecyl trimethyl ammonium Bromide (CTAB, 99%), methylene blue (MB) and 5,5′-dithiobis-(2-nitrobenzoic acid) (DTNB) were all purchased from Aladdin. Ammonium Hydroxide (NH_3_·H_2_O) and ethanol were purchased from Sinopharm Chemical Reagent Co., Ltd. Cell Counting Kit-8 (CCK-8), Calcein-AM/propidium iodide (PI), 4',6-diamidino-2-phenylindole (DAPI), 2,7-Dichlorodihydrofluorescein diacetate (DCFH-DA), Annexin V-FITC Apoptosis Detection Kit were obtained from Beyotime Biotech (China). Penicillin-Streptomycin and Dulbecco’s modified Eagle medium (DMEM) were purchased from HyClone (USA). Fetal bovine serum was purchased from Gibco (USA). All chemicals in this work were used as received without further purification. Deionized water (18.2 MΩ·cm, 25 °C) was used in all experiments, and all solutions were freshly prepared for immediate use in each experiment.

**Synthesis of FeOOH nanospindles:**

FeOOH nanospindles were synthesized by a hydrothermal method according to previous research^1^. Briefly, 2.7 g FeCl_3_·6H_2_O and 2.5 g CTAB were dispersed in 100 mL H_2_O. The mixture was then transferred into a 250 mL glass flask. The flask was transferred into an oven, heated to 60 °C and maintained for 12 h. After cooling down to room temperature, the product was centrifuged, washed with distilled water and ethanol, and finally dispersed in ethanol for further usages.

**Synthesis of** **FeOOH&mSiO_2_ Janus nanoparticles:**

For the fabrication of FeOOH&mSiO_2_ Janus nanoparticles with sushi-like structure, 8 mg of the obtained FeOOH nanoparticles were dispersed in 10 mL of deionized water followed by the addition of 9.6 mM (35 mg) CTAB. After ultrasonication for 30 minutes, the solution was transferred to 313 K oil bath under constant magnetic stirring of 500 rpm. NH_3_·H_2_O (5% (v/v)) was then added to the solution, followed by the dropwise addition of 80 μL TEOS. The above solution was allowed to react for 1 h. The FeOOH&mSiO_2_ Janus nanoparticles were centrifuged and washed by ethanol for 3 times to remove excessive CTAB. Subsequently, the products were redispersed in 25 mL of ethanol for further use.

**Synthesis of FeOOH@SiO_2_ core@shell nanoparticles:**

FeOOH@SiO_2_ core@shell nanoparticles with nonporous SiO_2_ shell were synthesized with a modified method according to a pervious research^2^. In brief, 32 mg of FeOOH nanospindles (~ 400 nm in diameter) were treated with 0.1 M HCl aqueous solution (50 mL) by ultrasonication. After the treatment for 10 min, FeOOH nanospindles were separated and washed with deionized water, and then homogeneously dispersed in the mixture of ethanol (40 mL), deionized water (10 mL) and concentrated ammonia aqueous solution (0.5 mL, 28 wt%), followed by the addition of 100 μL TEOS. After stirring at room temperature for 6 h, the FeOOH@SiO_2_ nanoparticles were separated and washed with ethanol and water. Subsequently, products were redispersed in 20 mL ethanol for further use.

**Synthesis of FeOOH@mSiO_2_ core@shell nanoparticles:**

FeOOH@mSiO_2_ core@shell nanoparticles with radially orientated mesoporous shell. were synthesized with a modified Stöber method^3^. Typically, 16 mg of the obtained FeOOH nanoparticles was added into the solution containing 48 mL of water, 48 mL of ethanol, 240 mg of CTAB, and 0.48 mL of ammonia aqueous solution (28 wt %). After stirring for 1 h, 0.4 mL of TEOS was added dropwise with continuous stirring and the reaction proceeded for 4 h to obtain the core@shell FeOOH@mSiO_2_ nanoparticles. The products were collected by centrifugation, washed with ethanol and water, and dispersed in 20 mL of ethanol.

**Synthesis of FeOOH&Periodic mesoporous organosilicon (PMO) Janus nanoparticles**

The synthesis of FeOOH&PMO Janus nanoparticles with anisotropic growth of cubic PMO compartment was based on the Stöber method. Typically, 1 mg of the obtained FeOOH nanoparticles was added into a solution containing 75 mL of water, 150 mg of CTAB and 1.8 mL of ammonia aqueous solution (28 wt%). After being stirred for 30 min, 0.1 mL of BTEE was added dropwise with continuous stirring, and the reaction was left to proceed for 3 h. Then, FeOOH&PMO Janus mesoporous nanocomposites were collected by centrifugation, washed with ethanol and water, and dispersed in ethanol.

**Synthesis of Au nanorods**

Au nanorods were synthesized by a bi-surfactant seeded growth strategy according to previous report^4^. The seed solution for gold NR growth was prepared as follows: 5 mL of 0.5 mM HAuCl_4_ was mixed with 5 mL of 0.2 M CTAB solution. Then, 0.6 mL of fresh 0.01 M NaBH_4_ was diluted to 1 mL with water and was then injected to the Au(III)-CTAB solution under vigorous stirring (1200 rpm). The solution color changed from yellow to brownish yellow and the stirring was stopped after 2 min. The seed solution was aged at room temperature for 30 min before use. To prepare the growth solution, 7.0 g (0.037 M in the final growth solution) of CTAB and 1.234 g of sodium oleate were dissolved in 250 mL of warm water (~50 °C). The solution was allowed to cool down to 30 °C and 18 mL 4 mM AgNO_3_ solution was added. The mixture was kept undisturbed at 30 °C for 15 min after which 250 mL of 1 mM HAuCl_4_ solution was added. The solution became colorless after 90 min of stirring (700 rpm) and 1 mL volume of HCl (37 wt. % in water, 12.1 M) was then introduced to adjust the pH to about 1. After another 15 min of slow stirring at 400 rpm, 1.25 mL of 0.064 M ascorbic acid (AA) was added and the solution was vigorously stirred for 30 s. Finally, 1 mL of seed solution was injected into the growth solution. The resultant mixture was stirred for 30 s and left undisturbed at 30°C for 12 h for NR growth. The final products were isolated by centrifugation at 12000 rpm for 20 mins followed by removal of the supernatant. Then the precipitate was dispersed in DIW of same volume for further use.

**Synthesis of Bi_2_S_3_ nanorods**

Bi_2_S_3_ nanorods were synthesized by a hydrothermal method according to a previous report^5^. Briefly, 0.498 g bismuth nitrate pentahydrate was added to 70 mL ethylene glycol solution with constant stirring. After vigorous stirring for 30 min, 0.5 g PVP was added to the solution and stirred for 30 min. 2 mL deionized H_2_O containing Na_2_S·9H_2_O (0.36 g) was added to the mixture dropwise and the mixture turned to black quickly. The solution was transferred into a 100 mL Teflon-lined autoclave, sealed, and heated up to 180 °C for 2 h in an electric furnace. Afterward, the autoclave was cooled naturally to room temperature, and the final products were collected by centrifugation and washed three times with ethanol and distilled water, respectively, to remove any possible remnants. The product was dispersed in ethanol for further use.

**Surface curvature-induced oriented self-assembly of mSiO_2_ nanorods on various kinds of premade nanorods/nanospindles:**

The surface curvature-induced oriented assembly of mSiO_2_ nanorods on various other kinds of pre-made nanorods/nanospindles was the same as the synthesis method of sushi-like structured FeOOH&mSiO_2_ Janus nanoparticles.

**Fenton reaction catalytic activity experiments.**

The Fenton reaction catalytic activity of FeOOH&mSiO_2_ Janus nanoparticles were detected using methylene blue (MB) as the substrate in the presence of H_2_O_2_ and GSH. FeOOH&mSiO_2_ Janus nanoparticles (125 µg/mL), GSH (0, 1, 2.5, 5, 10 mM), H_2_O_2_ (10 mM) and MB (10 µg/mL) were added into 1 mL of PBS (pH 5.4). The absorption peak of MB at 665 nm was recorded by a microplate reader.

**Drug loading and release**

A 1 mL amount of DOX was mixed with 1 mL FMS at different DOX : FMS ratios (2:3, 1:1, and 2:1, w/w), then the mixture was stirred at room temperature for 24 h. After that, the DOX-loaded product was separated by centrifugation, and the unloaded DOX in the supernatant was measured by UV/vis absorption at 480 nm. The drug loading efficiency and loading capacity were calculated as the following formulas:

loading efficiency = 100% × (total DOX – unloaded DOX) / total DOX (1)

loading capacity = (total DOX – unloaded DOX) / total FMS (2)

The drug release experiments were performed in PBS with different pH values (pH 7.4 and 5.4). Typically, 0.5 mg of DOX-loaded FMS (FMS-DOX) was re-dispersed in 2 mL of PBS in a tube, and then shaking at 37 °C. At given time points, the solution was centrifuged and 1 mL of the supernatant was collected for analysis with UV/vis absorption at 480 nm, and replaced with fresh PBS with the same volume and the same pH value.

**Cell culture.**

Murine breast cancer cell line (4T1 cells) was purchased from cell bank of Chinese academy of science (Shanghai, China). The cells were cultured in standard Penicillin-Streptomycin and Dulbecco’s modified Eagle medium (DMEM) supplemented with 10% (v/v) FBS, 100 μg/mL streptomycin and 100 U/mL penicillin at 37 °C in a humidified incubator with 5% CO_2_.

***In-vitro* cell uptake of** **DOX-loaded FeOOH&mSiO_2_.**

The intracellular endocytosis of FeOOH&mSiO_2_ Janus nanocomposites was investigated by confocal laser scanning microscopy (CLSM) and flow cytometry. For CLSM observation, 4T1 cells were seeded in the CLSM-exclusive culture dishes (105 cells per dish) and incubated for 24 h. Then, DOX-loaded FeOOH&mSiO_2_ nanocomposites (200 µg/mL) were added in to the culture media. After co-incubation for 0, 4, 8, 12 h, the cells were washed with PBS. Then, the cells were stained with 4',6-diamidino-2-phenylindole (DAPI) for 10 mins and imaged by CLSM. Moreover, the cells were collected by trypsin digestion and transferred into the test tube for the quantitative analyses of the fluorescence signal by flow cytometry.

***In-vitro* cytotoxicity evaluation.**

The cytotoxicity of the prepared FeOOH&mSiO_2_ nanocomposites was assessed using standard Cell Counting Kit-8 (CCK-8) assay. 4T1 cells were seeded in 96-well plates (105 cells/well) for 24 h. After that, the cells were incubated with fresh medium containing different concentrations of FeOOH&mSiO_2_ nanocomposites (200, 100, 50, 25, 12.5, 6.25, 3.125 μg/mL) with or without H_2_O_2_ (10 mM) and GSH (10 mM) for another 24 h. Then, the medium was discarded and the cells were washed with PBS. The mixture of CCK-8 and fresh culture medium was added into each well and incubated for 2 h. Finally, the cell viability was evaluated by measuring the absorbance at the wavelength of 450 nm.

***In-vitro* therapeutic efficiency.**

Confocal laser scanning microscopiy (CLSM) and flow cytometry were introduced to evaluate the in-vitro therapeutic efficacy of FeOOH&mSiO_2_ and DOX-loaded FeOOH&mSiO_2_ Janus nanocomposites. 4T1 cells were seeded in the CLSM-exclusive culture dishes (105 cells per dish) and incubated for 24 h. The cells were then co-incubated with FeOOH&mSiO_2_ or DOX-loaded nanocomposites (200 μg/mL) with or without H_2_O_2_ (10 mM) and GSH (10 mM) in the DMEM culture medium (pH 5.4). After 12 h, the cells were washed with PBS for three times, stained with Calcein-AM/PI after different treatments for CLSM observation. The quantitative analysis of the Cell apoptosis was determined by flow cytometry after co-incubation with Annexin V-FITC/PI apoptosis detection kit in dark for 20 min.

**Tumor model.**

All the animal experiments were approved by the Shanghai Science and Technology Committee and performed in agreement with the guidelines of the Department of Laboratory Animal Science, Fudan University. 4 ~ 6 weeks-old Female Balb/c mice were commercially supplied by Shanghai JSJ Laboratory Animal Co. Ltd. (Shanghai, China). 4T1 cells suspended in FBS (1 × 107 cells) were subcutaneously injected into the right back leg of mice. When the tumor volume has grown to ~ 100 mm^3^, the tumor-bearing mice were randomly divided into 4 groups (n = 3 for each group): control, FMS, DOX and FMS-DOX. Saline and its solution of FMS, DOX and FMS-DOX were intravenously injected to four groups of mice respectively at 0, 2^nd^, 5^th^, 9^th^, 12^th^, 16^th^, 19^th^ and 21^st^ day, and the body weights and tumor sizes of each group were monitored periodically. All mice were sacrificed and dissected at 21^st^ day, organ sections were stained with Hematoxylin and eosin (H&E) for observation.

**Measurements and characterization.**

Transmission electron microscopy (TEM) observations were acquired on JEM-2100F with an accelerating voltage of 200 kV equipped with a post-column Gatan imaging filter (GIF-Tridium). Scanning electron microscopy (SEM) images were taken using a Hitachi S-4800 ultrahigh resolution cold FEG with an in-lens electron optic operating at 20 kV. Nitrogen adsorption-desorption measurements were conducted to obtain information on the porosity. The measurements were conducted at 77 K with ASAP 2420 analyzer (USA). The UV/Vis spectra were recorded on Lambda 35 Perkin-Elmer. Confocal fluorescence images were obtained by an LSM 980 confocal laser scanning microscope (Carl Zeiss SMT Inc., USA). Flow cytometry analysis was performed by an Accuri C6 flow cytometer (BD Biosciences, USA).

# Supplementary Figures


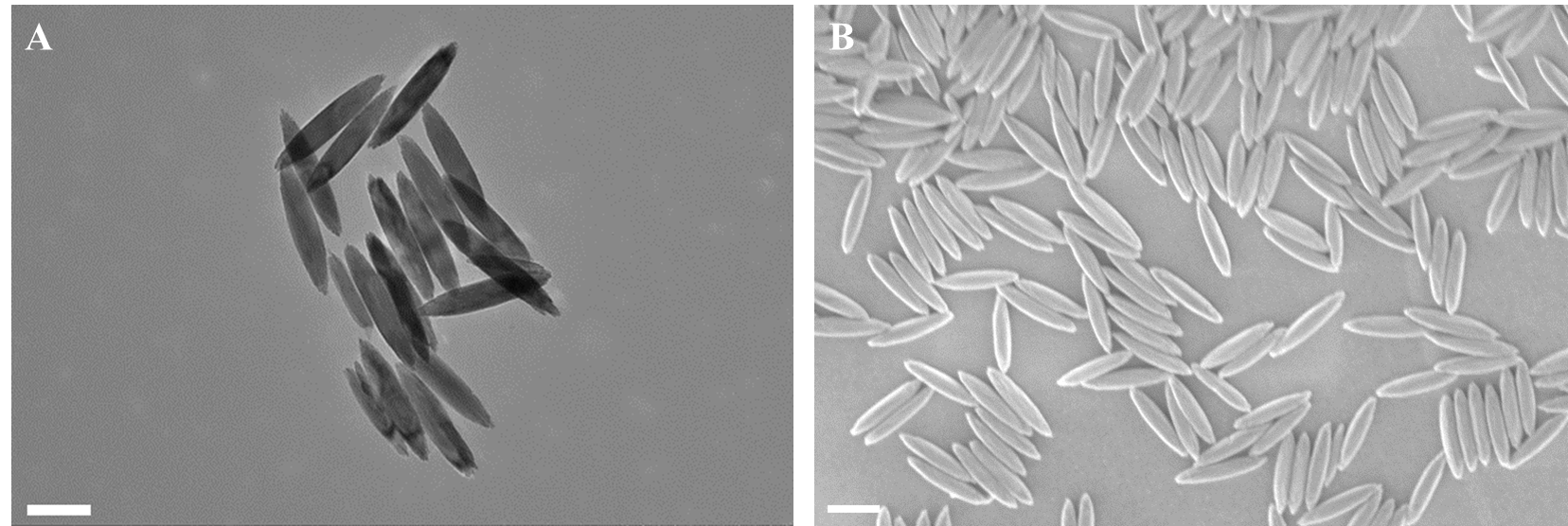


**Supplementary Figure 1.** (A) Transmission electron microscopy (TEM) and (B) scanning electron microscopy (SEM) images of FeOOH nanospindles. The FeOOH nanospindles are of uniform size and well dispersed. Scale bar: 200 nm.


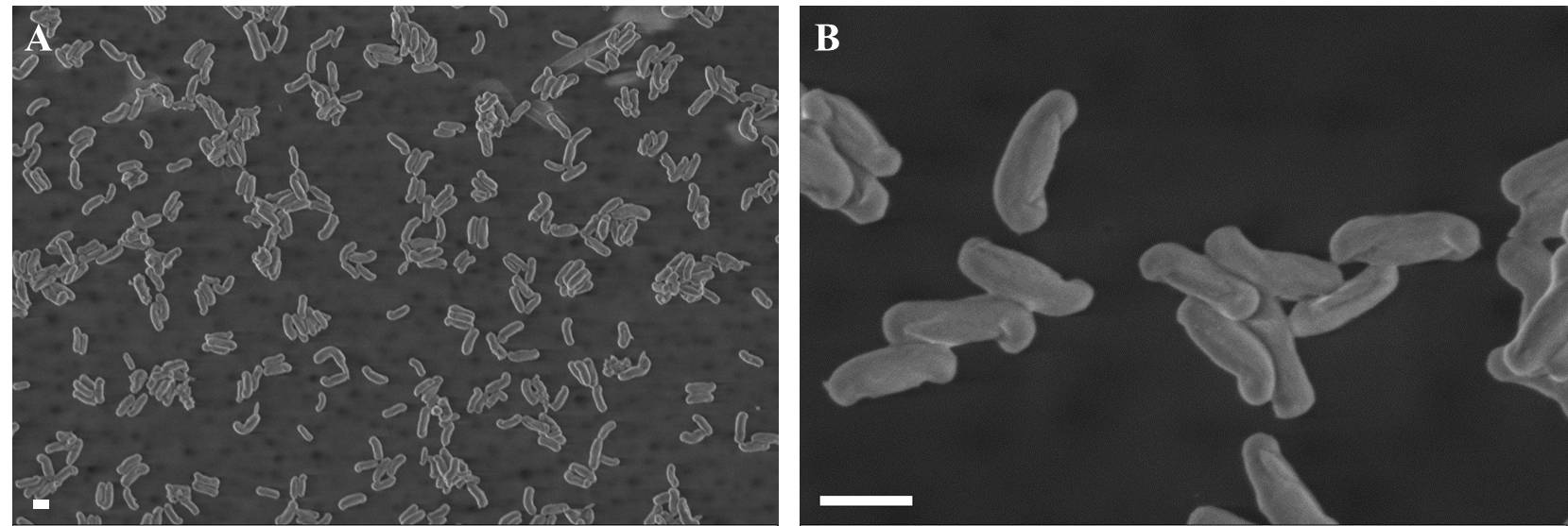


**Supplementary Figure 2.** Large area SEM images of FeOOH&mSiO_2_ Janus nanoparticles with different magnifications, indicating that the FeOOH&mSiO_2_ Janus nanoparticles are of uniform morphology and well dispersed. Scale bar: 200 nm.

**
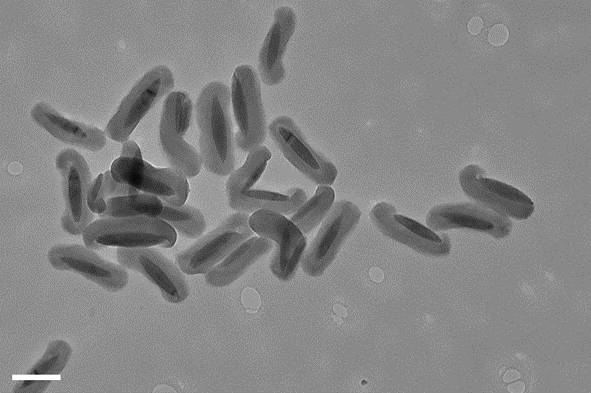
**

**Supplementary Figure 3.** TEM image of sushi-like Janus FeOOH&mSiO_2_ after being ultrasonicated for 30 minutes. The sushi-like structure was retained well even under harsh physical environment, which proved the superior structural stability of the sushi-like Janus structure. Scale bar: 200 nm.

**
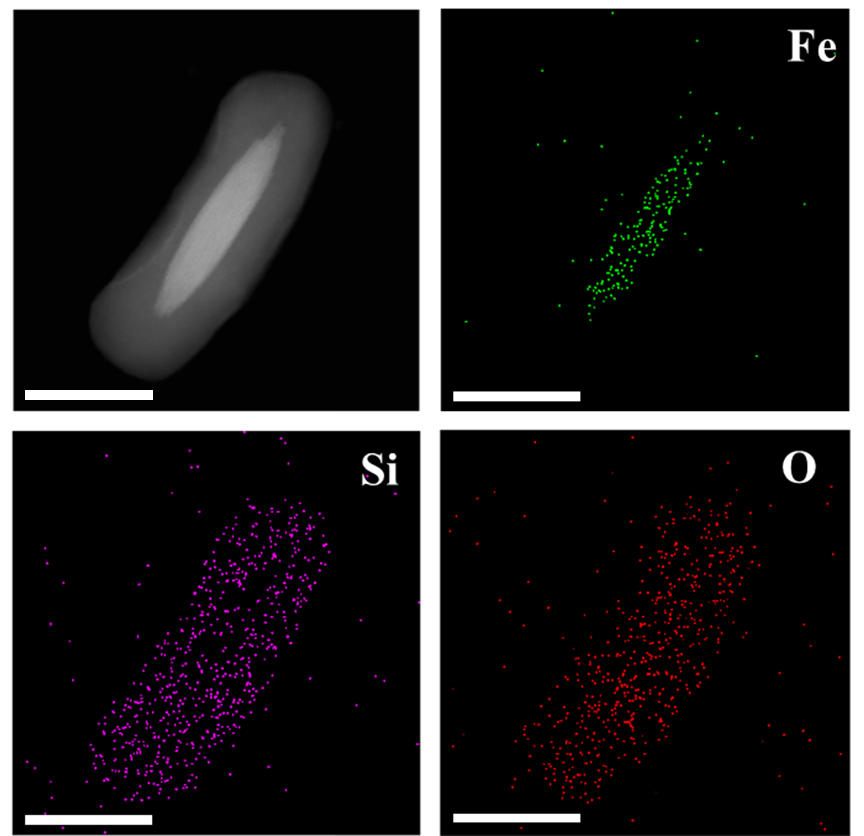
**

**Supplementary Figure 4.** Elemental mapping of Fe, Si and O in the FeOOH&mSiO_2_ Janus nanoparticles from a top-down view. The mSiO_2_ nanorod covers the long axis of FeOOH nanospindle, forming sushi-like Janus structure. Scale bar: 200 nm.

**
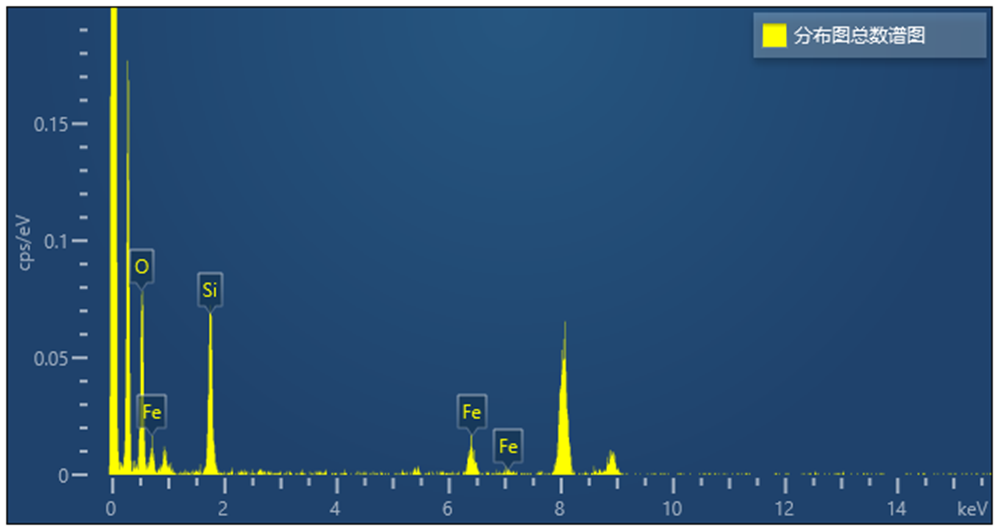
**

| **Element** | **k factor** | **Absorption correction** | **Wt%** | **Wt% sigma** | **Percentage of atom (%)** |
| --- | --- | --- | --- | --- | --- |
| **O** | 2.035 | 1.00 | 54.74 | 2.11 | 70.99 |
| **Si** | 1.000 | 1.00 | 33.20 | 1.84 | 24.53 |
| **Fe** | 1.129 | 1.00 | 12.06 | 1.17 | 4.48 |
| **Total** |  |  | 100.00 |  | 100.00 |

**Supplementary Figure 5.** Energy Dispersive Spectroscopy (EDS) pattern and data of FeOOH&mSiO_2_ Janus nanoparticle, indicating the existence of Si, Fe elements. Si/Fe atomic ratio is about 5:1.

**
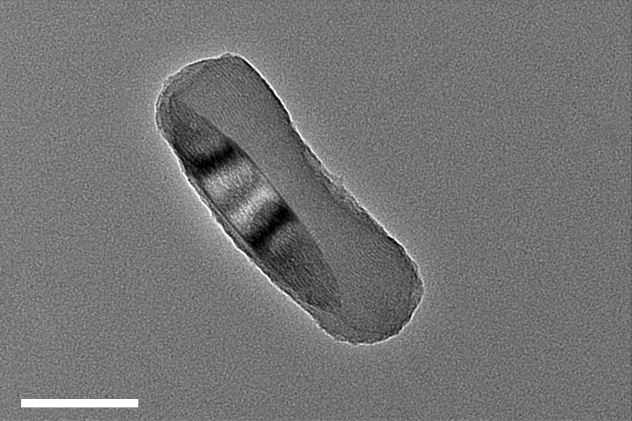
**

**Supplementary Figure 6.** High resolution-TEM image of FeOOH&mSiO_2_ Janus nanoparticles with different magnifications, the mesopores in the mSiO_2_ nanorod can be clearly seen to be parallel to the FeOOH nanospindle. Scale bar: 100 nm.

**
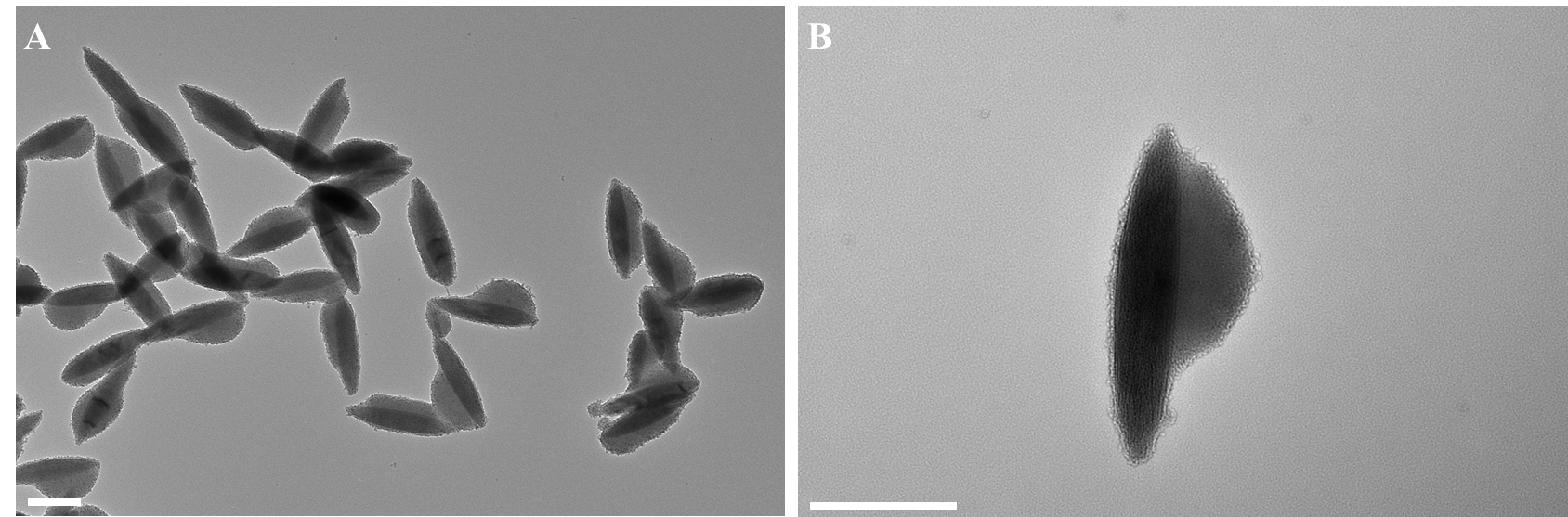
**

**Supplementary Figure 7.** TEM images of axe-like FeOOH&mSiO_2_ Janus nanoparticles synthesized with 1.4 mM CTAB with different magnifications. All conditions are fixed as the experiment section mentioned above except for the altered CTAB concentration. Scale bar: 200 nm.

**
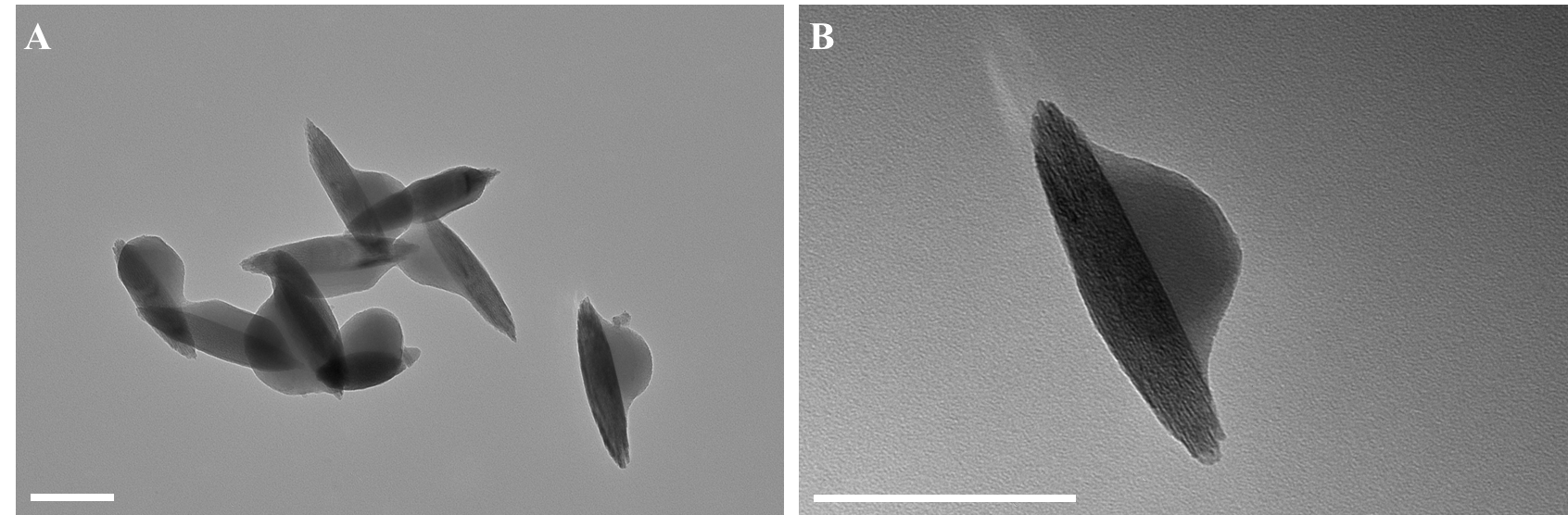
**

**Supplementary Figure 8.** TEM images of axe-like FeOOH&mSiO_2_ Janus nanoparticles synthesized with 2.7 mM CTAB with different magnifications. All conditions are fixed as the experiment section mentioned above except for the altered CTAB concentration. Scale bar: 200 nm.

**
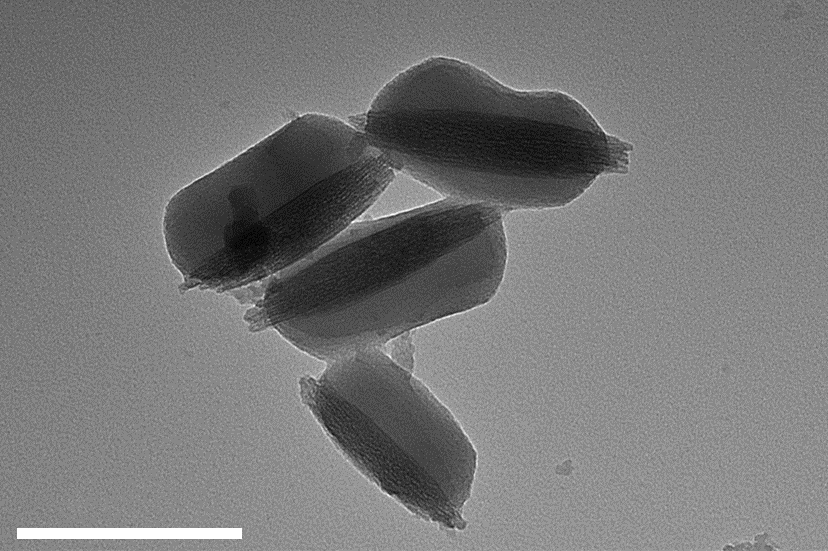
**

**Supplementary Figure 9.** TEM image of FeOOH&mSiO_2_ Janus nanoparticles synthesized with 6.9 mM CTAB. All conditions are fixed as the experiment section mentioned above except for the altered CTAB concentration. Scale bar: 200 nm.

**
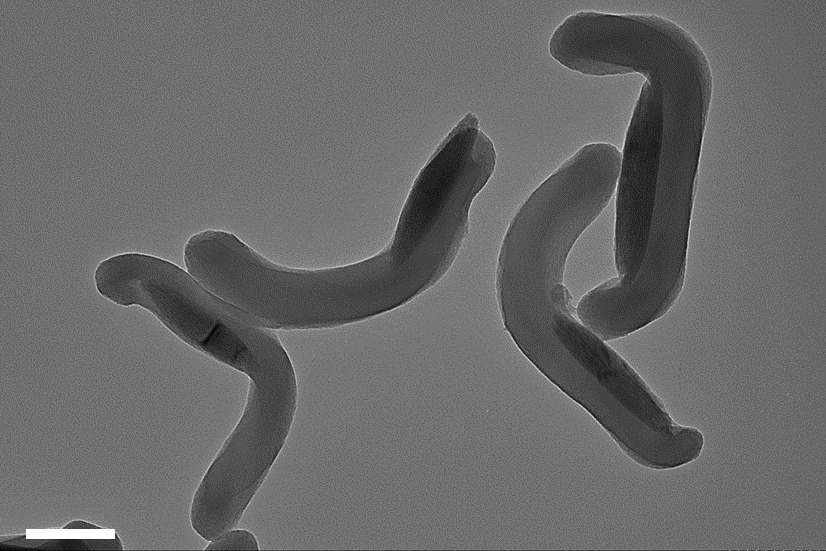
**

**Supplementary Figure 10.** TEM image of FeOOH&mSiO_2_ Janus nanoparticles synthesized with 13.7 mM CTAB. All conditions are fixed as the experiment section mentioned above except for the altered CTAB concentration. Scale bar: 200 nm.

**
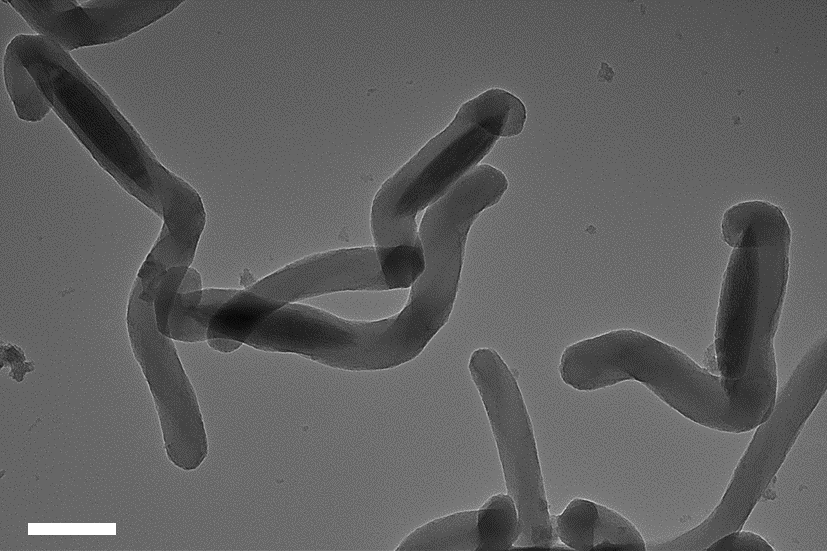
**

**Supplementary Figure 11.** TEM image of FeOOH&mSiO_2_ Janus nanoparticles synthesized with 20.6 mM CTAB. All conditions are fixed as the experiment section mentioned above except for the altered CTAB concentration. Scale bar: 200 nm.

**
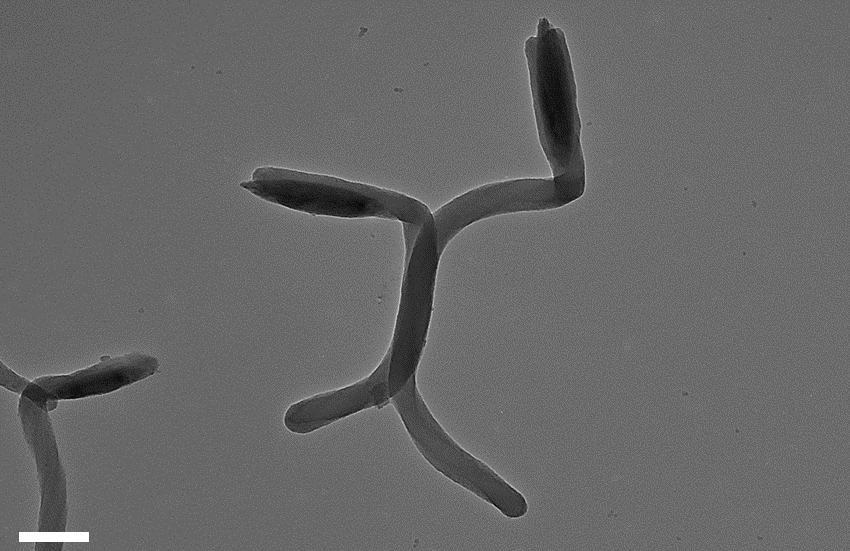
**

**Supplementary Figure 12.** TEM image of FeOOH&mSiO_2_ Janus nanoparticles synthesized with 27.4 mM CTAB. All conditions are fixed as the experiment section mentioned above except for the altered CTAB concentration. Scale bar: 200 nm.

**
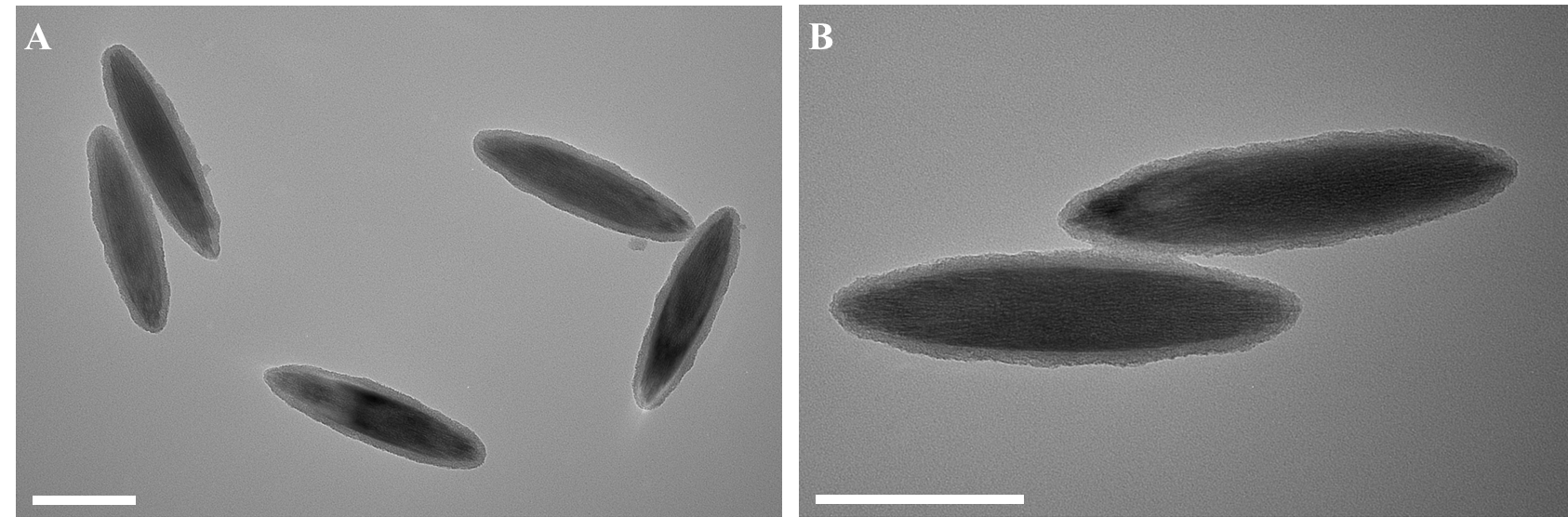
**

**Supplementary Figure 13.** TEM images of FeOOH@mSiO_2_ nanoparticles synthesized with 1% (v/v) NH_3_·H_2_O with different magnifications. All conditions are fixed as the experiment section mentioned above except for the altered amount of NH_3_·H_2_O. Scale bar: 200 nm.

**
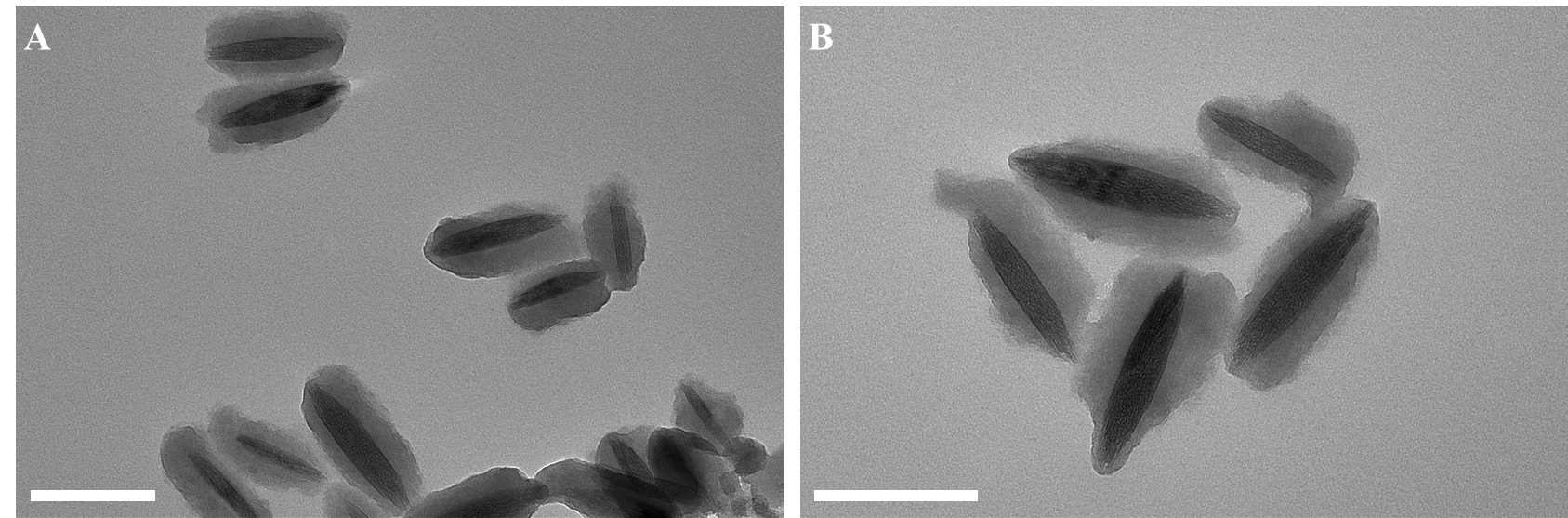
**

**Supplementary Figure 14.** TEM images of FeOOH&mSiO_2_ nanoparticles synthesized with 2% (v/v) NH_3_·H_2_O with different magnifications. All conditions are fixed as the experiment section mentioned above except for the altered amount of NH_3_·H_2_O. Scale bar: 200 nm

**
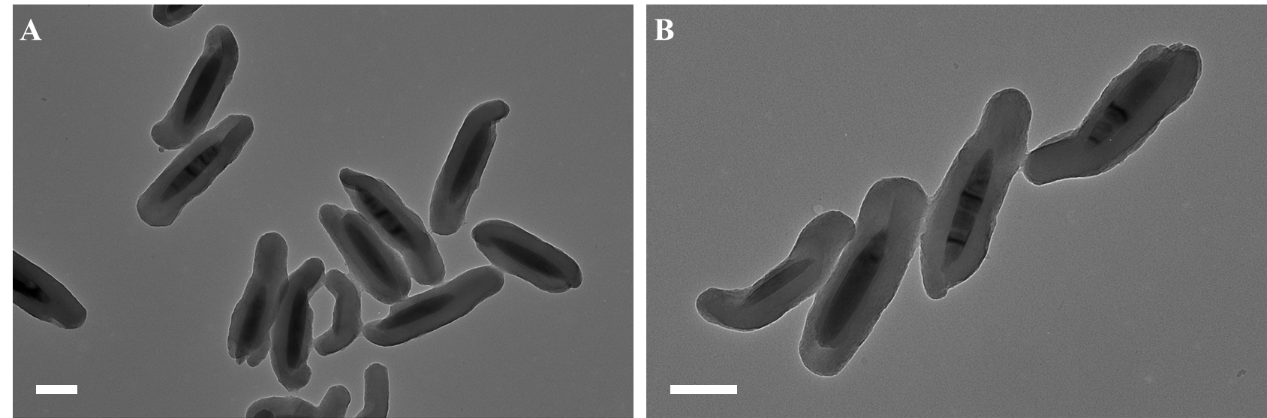
**

**Supplementary Figure 15.** TEM images of FeOOH&mSiO_2_ Janus nanoparticles synthesized with 3% (v/v) NH_3_·H_2_O with different magnifications. All conditions are fixed as the experiment section mentioned above except for the altered amount of NH_3_·H_2_O. Scale bar: 200 nm.

**
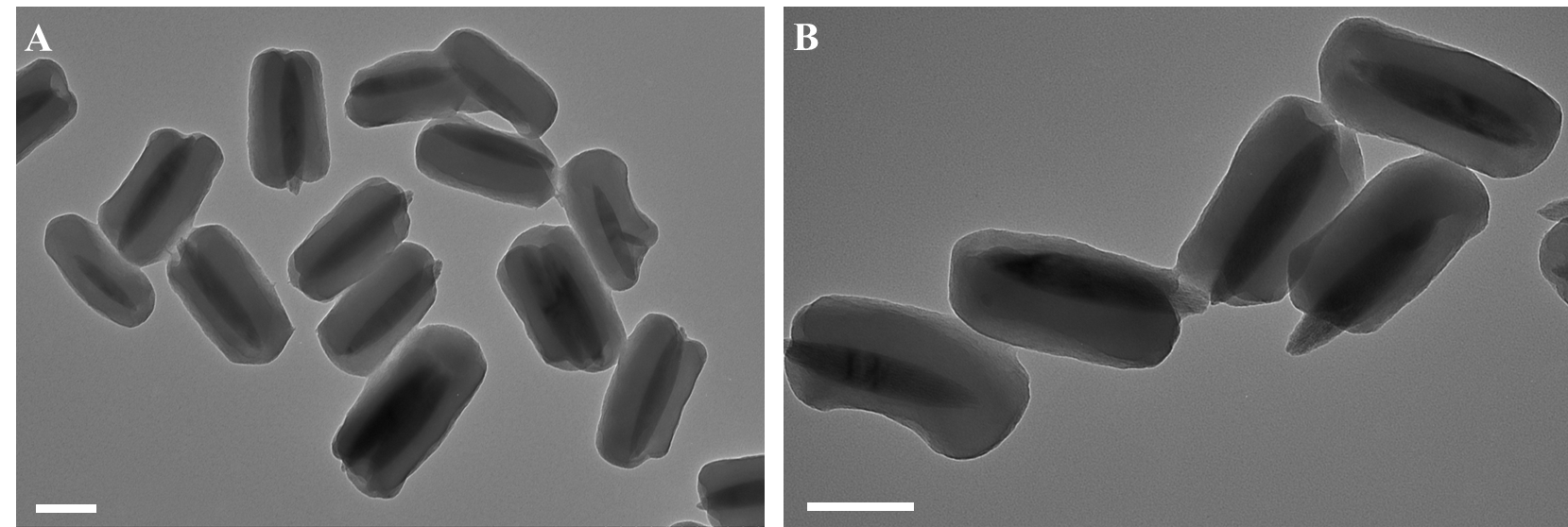
**

**Supplementary Figure 16.** TEM images of FeOOH&mSiO_2_ Janus nanoparticles synthesized with 4% (v/v) NH_3_·H_2_O with different magnifications. All conditions are fixed as the experiment section mentioned above except for the altered amount of NH_3_·H_2_O. Scale bar: 200 nm.

**
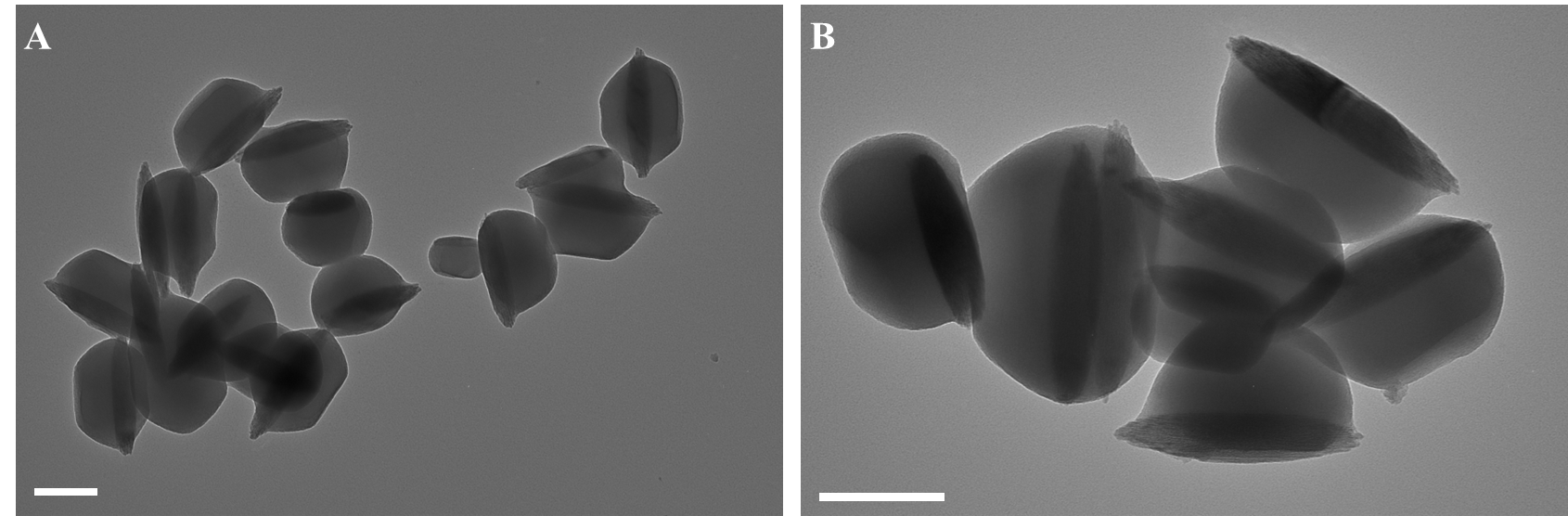
**

**Supplementary Figure 17.** TEM images of FeOOH&mSiO_2_ Janus nanoparticles synthesized with 8% (v/v) NH_3_·H_2_O with different magnifications. All conditions are fixed as the experiment section mentioned above except for the altered amount of NH_3_·H_2_O. Scale bar: 200 nm.

**
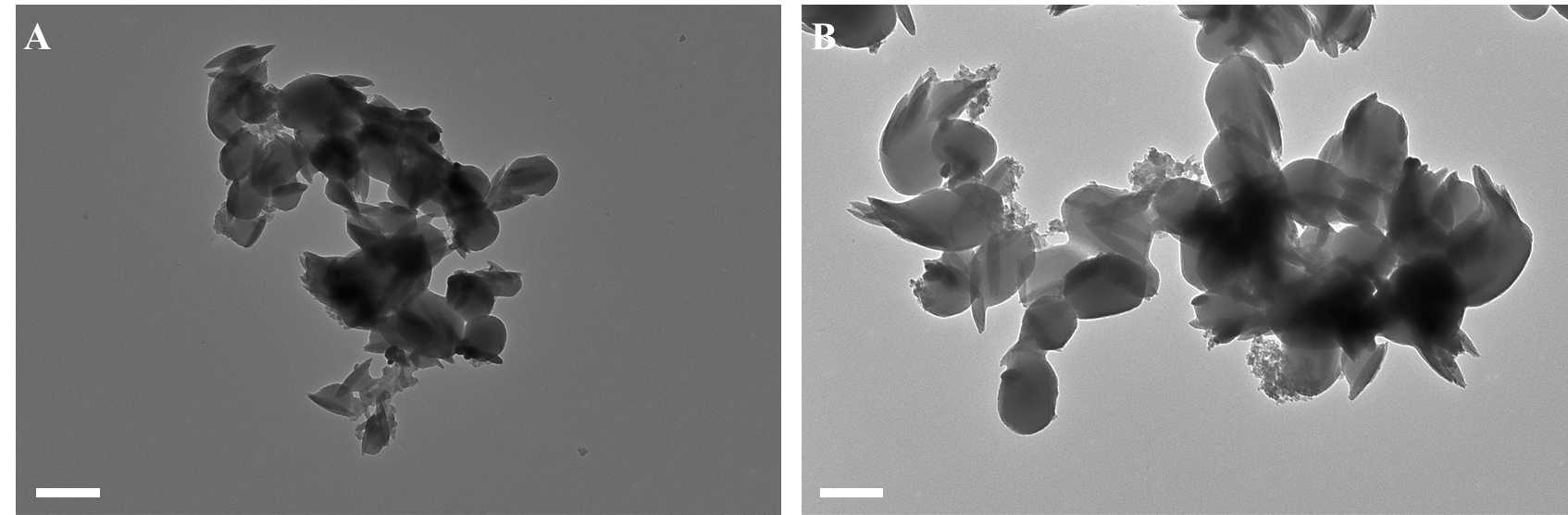
**

**Supplementary Figure 18.** TEM images of FeOOH&mSiO_2_ nanoparticles synthesized with 10% (v/v) NH_3_·H_2_O with different magnifications. All conditions are fixed as the experiment section mentioned above except for the altered amount of NH_3_·H_2_O. Scale bar: 200 nm.

**
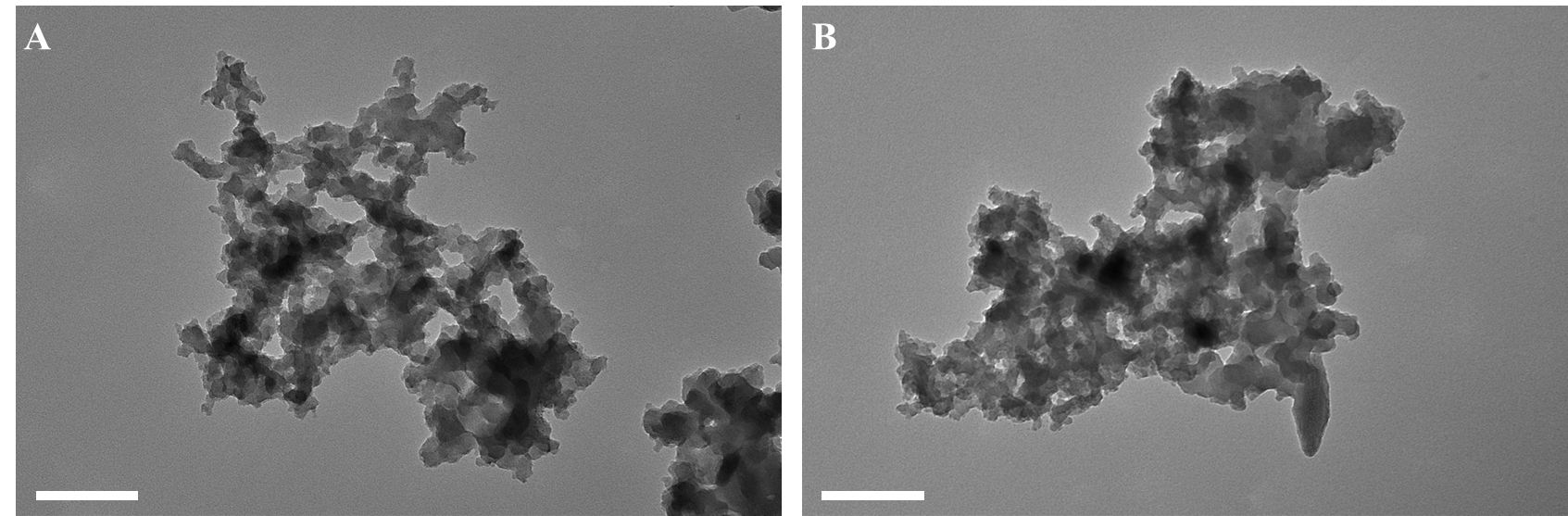
**

**Supplementary Figure 19.** TEM images of FeOOH&mSiO_2_ nanoparticles synthesized with 20% (v/v) NH_3_·H_2_O. All conditions are fixed as the experiment section mentioned above except for the altered amount of NH_3_·H_2_O. Scale bar: 200 nm.

**
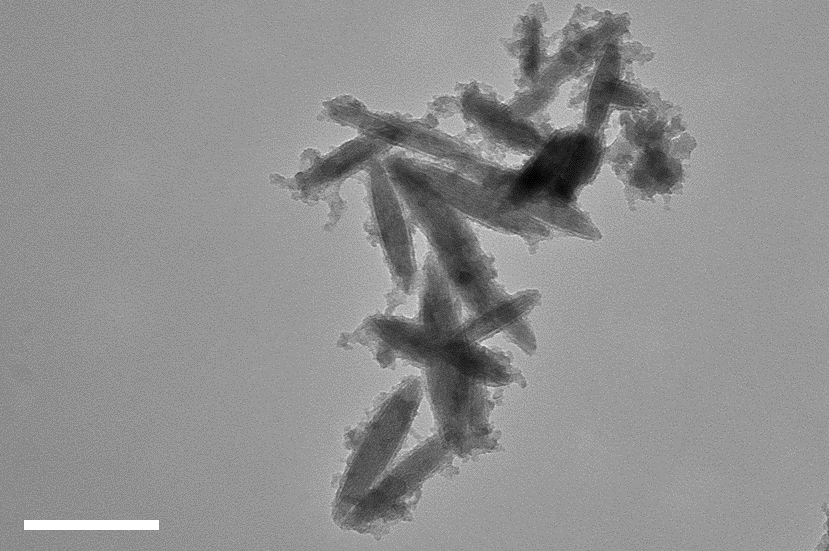
**

**Supplementary Figure 20.** TEM image of FeOOH&mSiO_2_ nanoparticles synthesized at ambient temperature (~298 K). All conditions are fixed as the experiment section mentioned above except for the altered synthetic temperature. Scale bar: 200 nm.

**
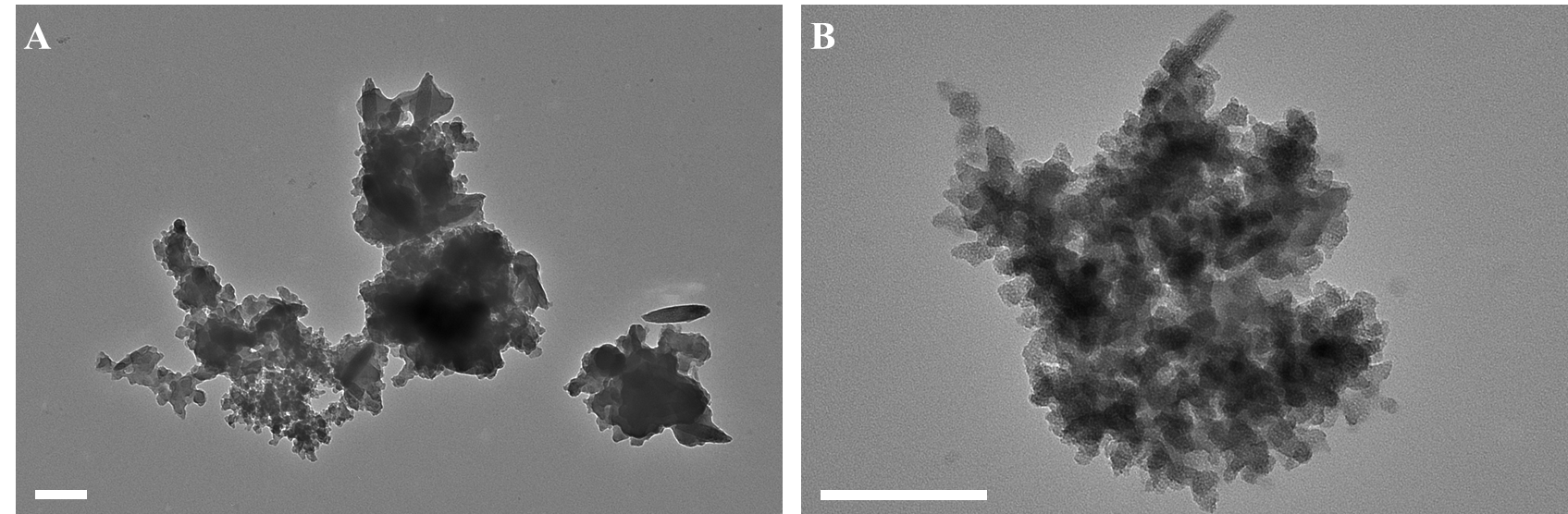
**

**Supplementary Figure 21.** TEM images of FeOOH&mSiO_2_ nanoparticles synthesized at 333 K with different magnifications. All conditions are fixed as the experiment section mentioned above except for the altered synthetic temperature. Scale bar: 200 nm.

**
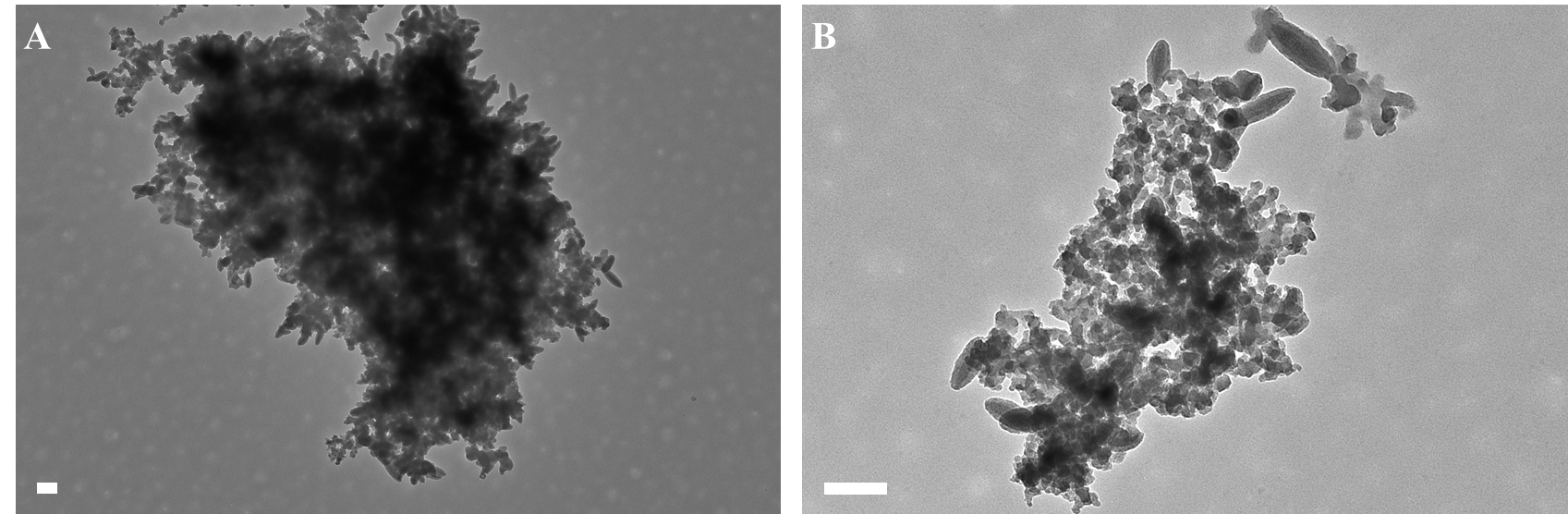
**

**Supplementary Figure 22.** TEM images of FeOOH&mSiO_2_ nanoparticles synthesized at 353 K with different magnifications. All conditions are fixed as the experiment section mentioned above except for the altered synthetic temperature. Scale bar: 200 nm.

**
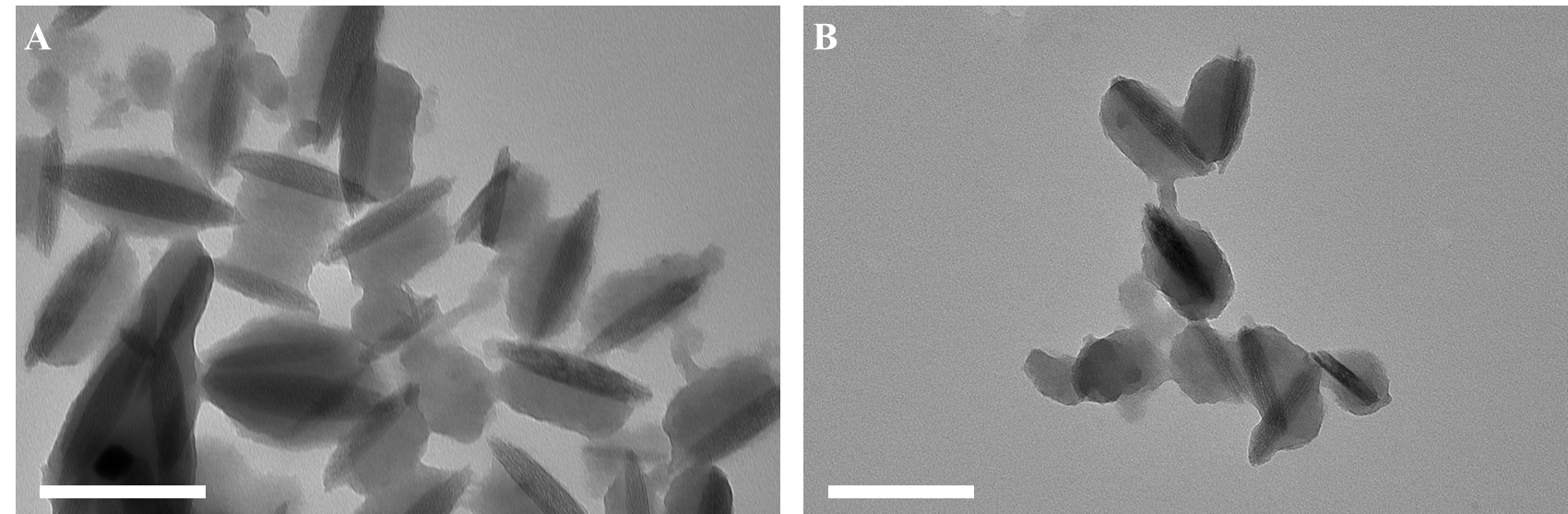
**

**Supplementary Figure 23.** TEM images of FeOOH&mSiO_2_ Janus nanoparticles synthesized with 40 μL TEOS with different magnifications. All conditions are fixed as the experiment section mentioned above except for the altered amount of TEOS. Scale bar: 200 nm.

**
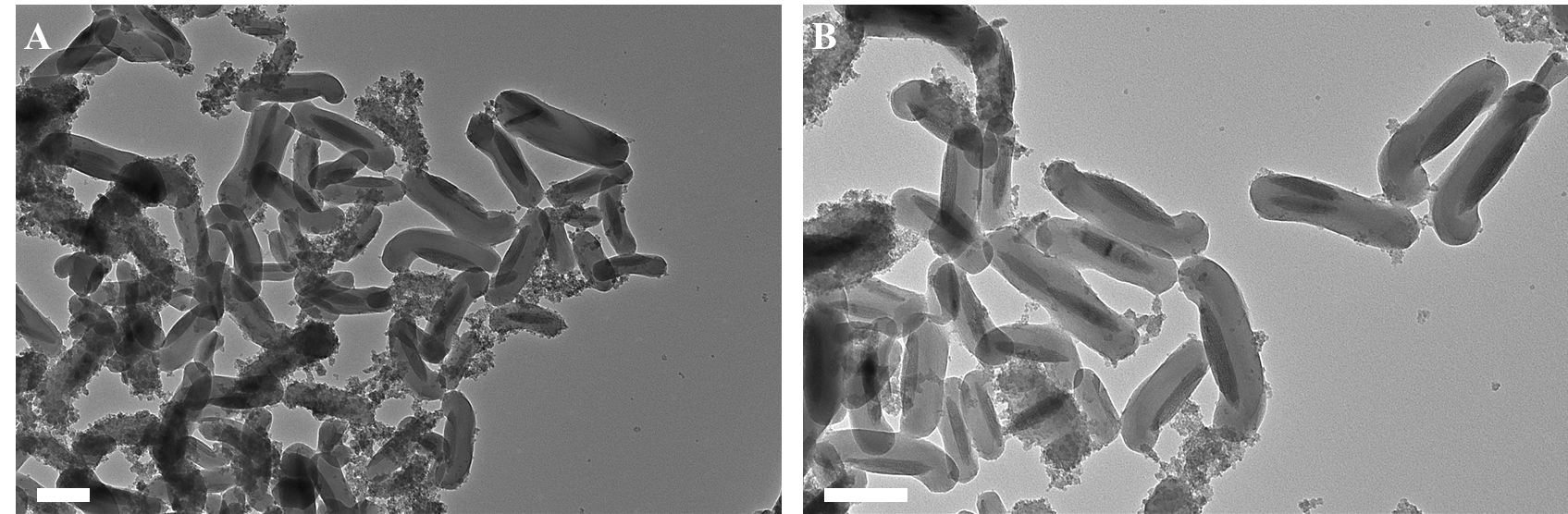
**

**Supplementary Figure 24.** TEM images of FeOOH&mSiO_2_ Janus nanoparticles synthesized with 120 μL TEOS with different magnifications. All conditions are fixed as the experiment section mentioned above except for the altered amount of TEOS. Scale bar: 200 nm.

**
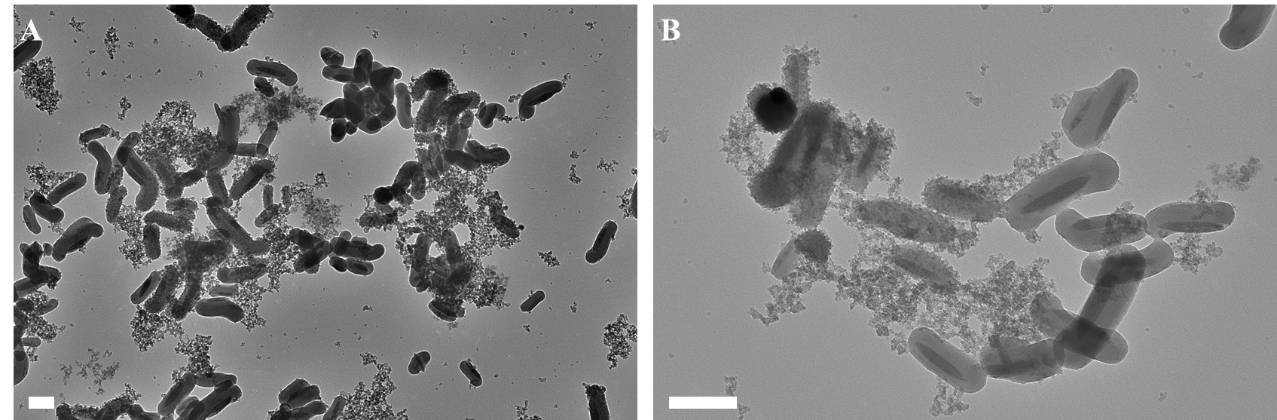
**

**Supplementary Figure 25.** TEM images of FeOOH&mSiO_2_ Janus nanoparticles synthesized with 160 μL TEOS with different magnifications. All conditions are fixed as the experiment section mentioned above except for the altered amount of TEOS. Scale bar: 200 nm.

**
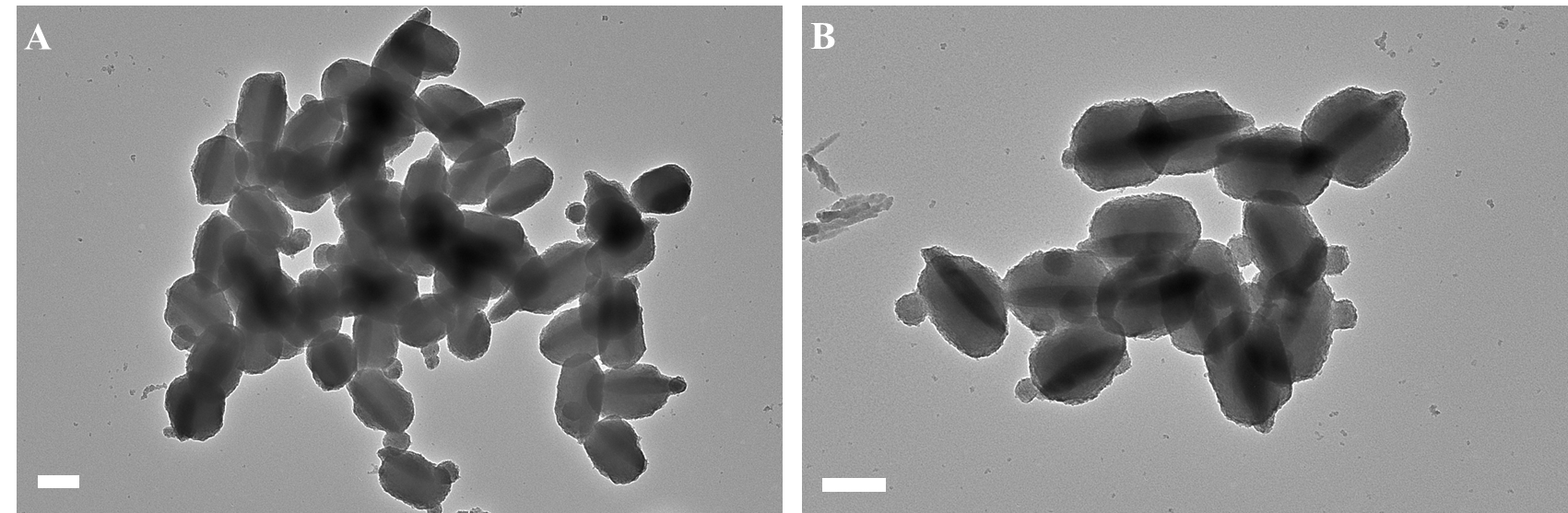
**

**Supplementary Figure 26.** TEM images of FeOOH&mSiO_2_ Janus nanoparticles synthesized with 200 μL TEOS with different magnifications. All conditions are fixed as the experiment section mentioned above except for the altered amount of TEOS. Scale bar: 200 nm.

**
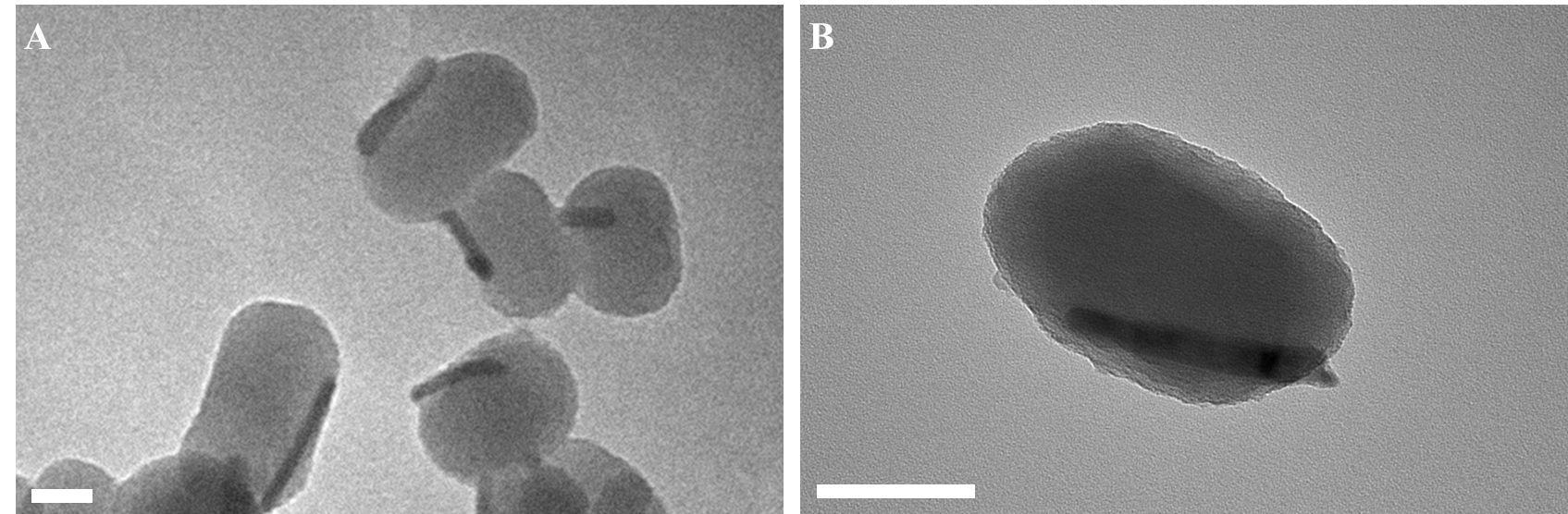
**

**Supplementary Figure 27.** TEM images of Bi_2_S_3_&mSiO_2_ Janus nanoparticles with different magnifications. Scale bar: 100 nm.

**
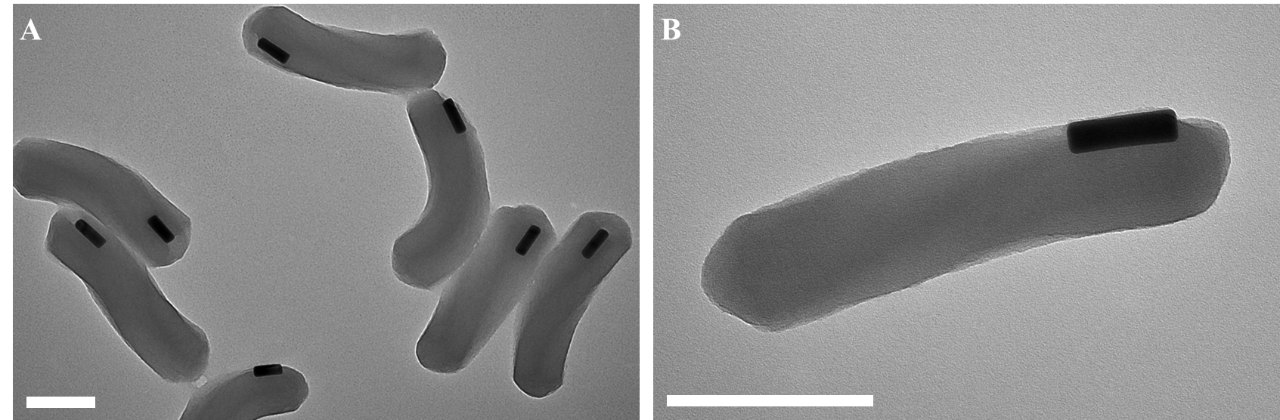
**

**Supplementary Figure 28.** TEM images of Au nanorod&mSiO_2_ Janus nanoparticles with different magnifications. Scale bar: 200 nm.

**
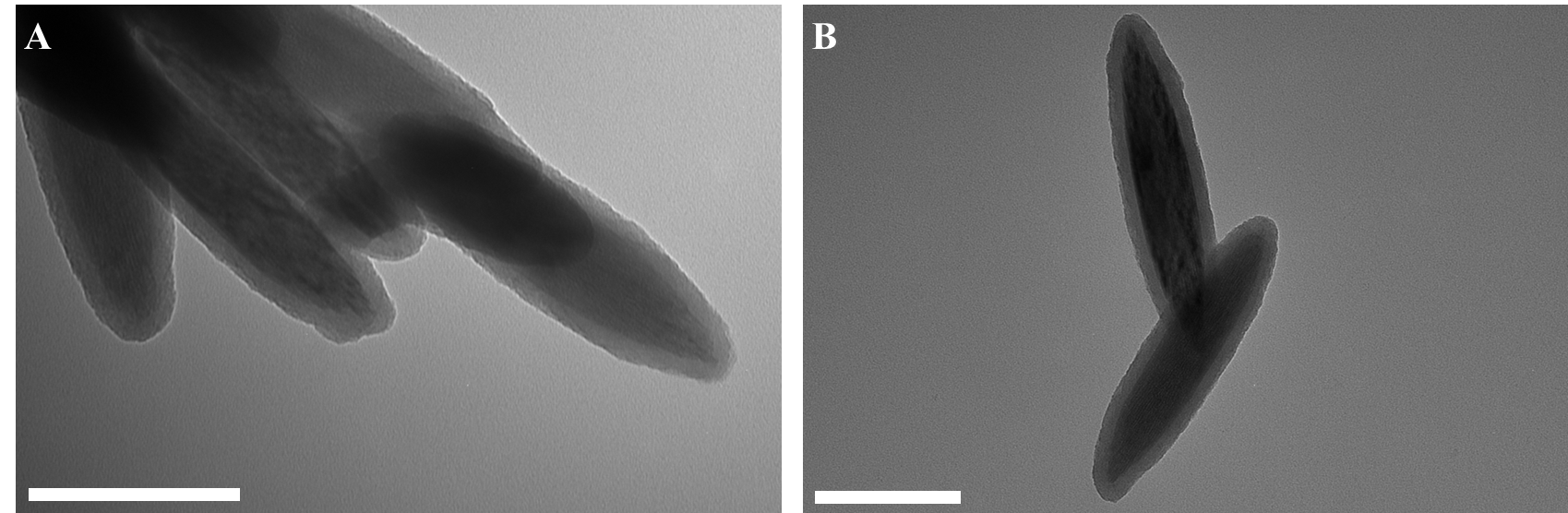
**

**Supplementary Figure 29.** TEM images of FeOOH&nSiO_2_ core@shell nanoparticles with different magnifications, indicating that the growth of nonporous silica on FeOOH nanospindle exhibits no long-axis selectivity, but covering the entire FeOOH nanospindle. Scale bar: 200 nm.

**
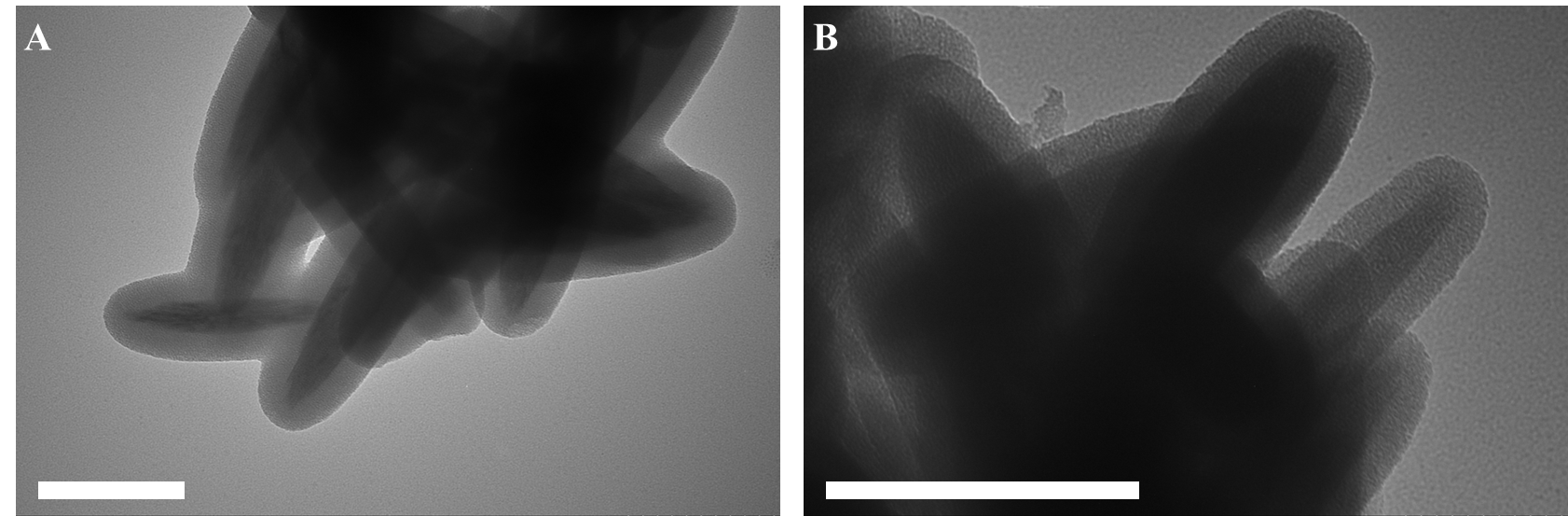
**

**Supplementary Figure 30.** TEM images of FeOOH&radial porous SiO_2_ core@shell nanoparticles with different magnifications, indicating that the growth of radial orientated mesoporous silica on FeOOH nanospindle exhibits no long-axis selectivity, but covering the entire FeOOH nanospindle. Scale bar: 200 nm.

**
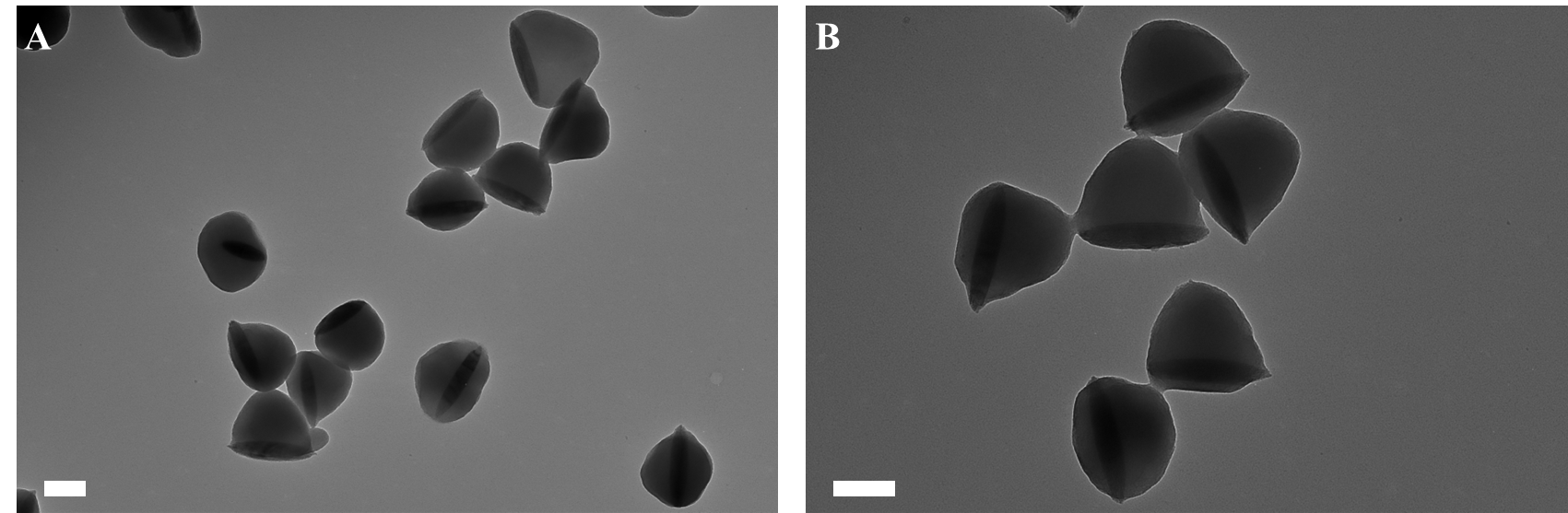
**

**Supplementary Figure 31.** TEM images of FeOOH&PMO Janus nanoparticles with different magnifications. The growth of PMO with cubic mesostructure on FeOOH nanospindle still exhibits long-axis selectivity, forming sushi-like structure, which further demonstrate the vital role of ordered mesostructure in long-axis selectivity. Scale bar: 200 nm.

**
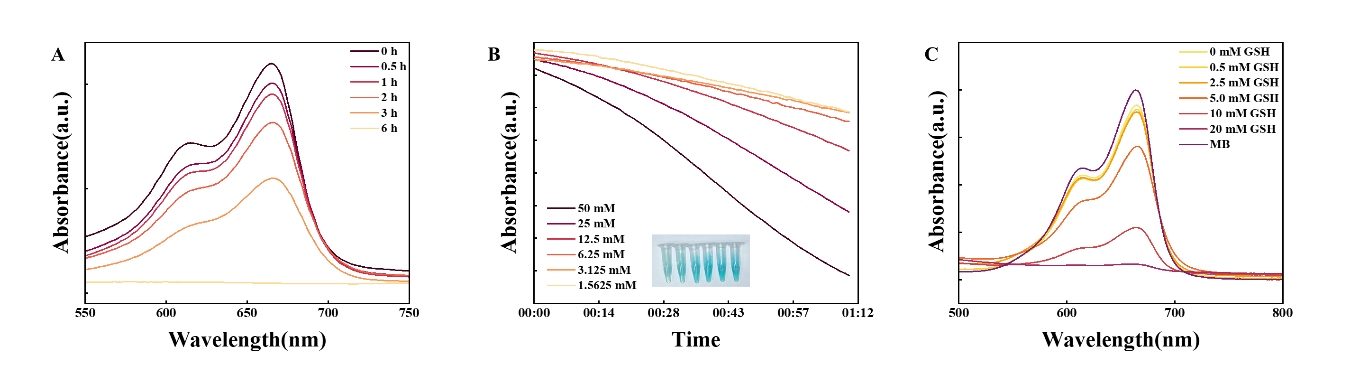
**

**Supplementary Figure 32.** (A) UV-vis spectra of methylene blue (MB) degradation at fixed time points under the catalysis of 125 μg/mL FMS with GSH (10 mM) and H_2_O_2_ (10 mM). (B) Time-dependent absorbance changes at 665 nm of the degradation of MB under the catalysis of 125 μg/mL FMS with GSH (10 mM) and different concentrations of H_2_O_2_ (1.5625-50 mM). Inset picture is the optical photograph of samples treated with different concentrations of H_2_O_2_ in an absorbance-ascending order. (C) UV-vis spectra of MB degradation under the catalysis of 125 μg/mL FMSs with H_2_O_2_ (10 mM) and GSH with different concentrations (0-20 mM). MB concentration: 10 μg/mL, pH: 5.4. All conditions were fixed as experimental section except for altered one.


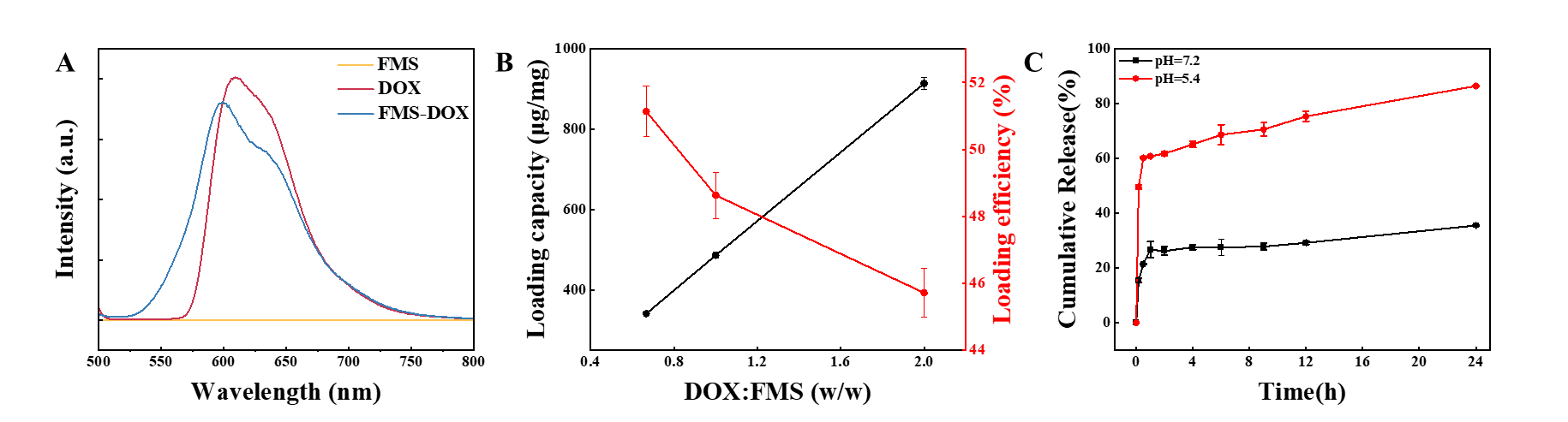


**Supplementary Figure 33.** DOX-loading and release profile of FMS-DOX. (A) Fluorescent spectroscopy of pristine FMS, DOX and FMS-DOX. (B) DOX-loading capacity and loading efficiency of FMS at different DOX/FMS ratios (2:3, 1:1 and 2:1). (C) DOX-release profile of FMS-DOX in neutral (7.2) and weak acidic (5.4) environment. Data are expressed as mean standard ± errors (n = 3).

**
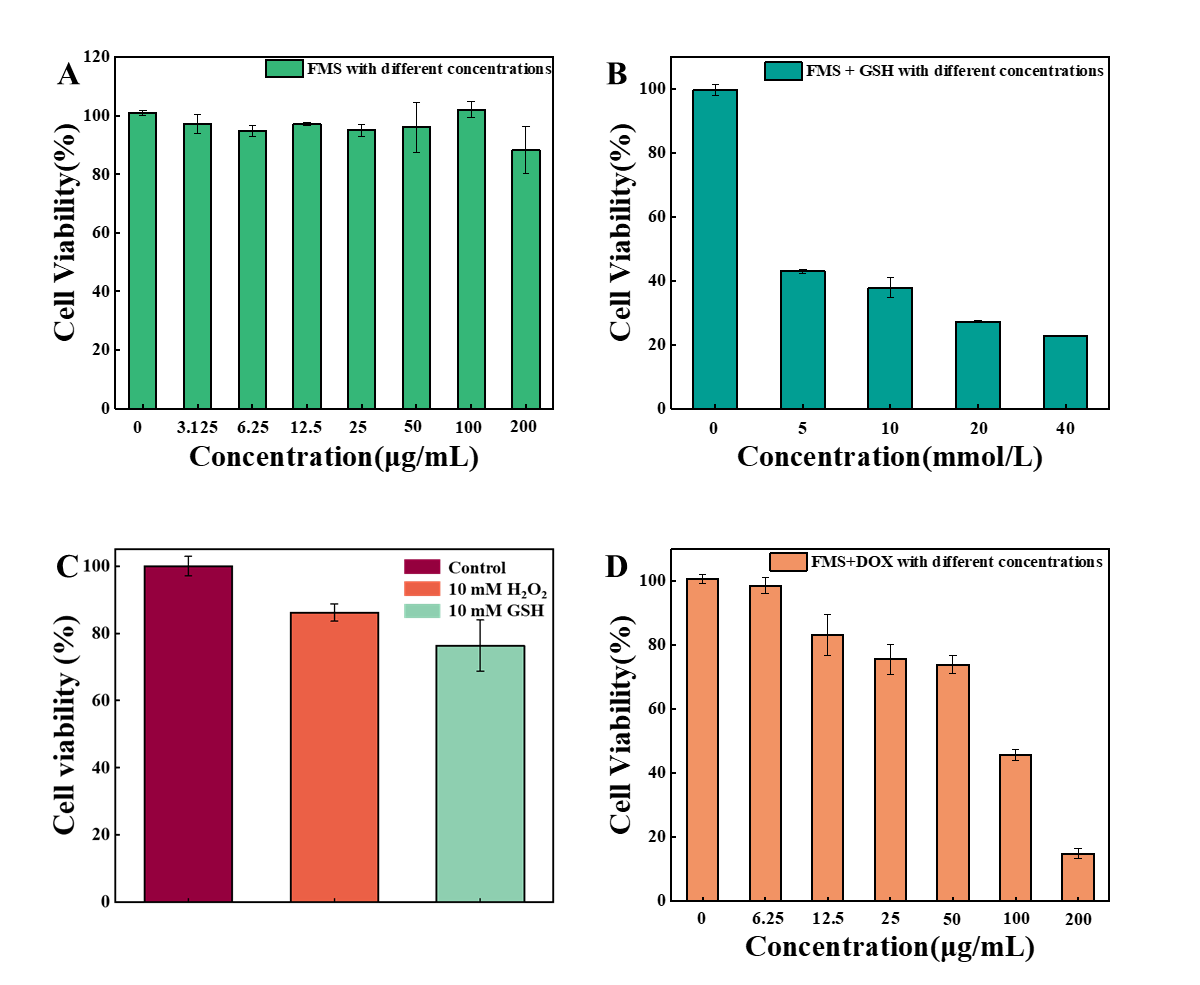
**

**Supplementary Figure 34.** (A) Cytotoxicity of pristine FMS in different concentrations (0-200 μg/mL). pH: 7.2. (B) In vitro therapeutic efficiency of FMS with 10 mM H_2_O_2_ and different concentrations of GSH (0-40 mM). pH: 5.4. (C) Cell viability of 4T1 cells with 10 mM GSH or 10 mM H_2_O_2_. pH: 7.2. (D) *In vitro* therapeutic efficiency of FMS-DOX with 10 mM GSH and 10 mM H_2_O_2_. pH: 5.4. Data are expressed as mean standard ± errors (n = 3). All conditions were fixed as experimental section mentioned above except for the alter one.


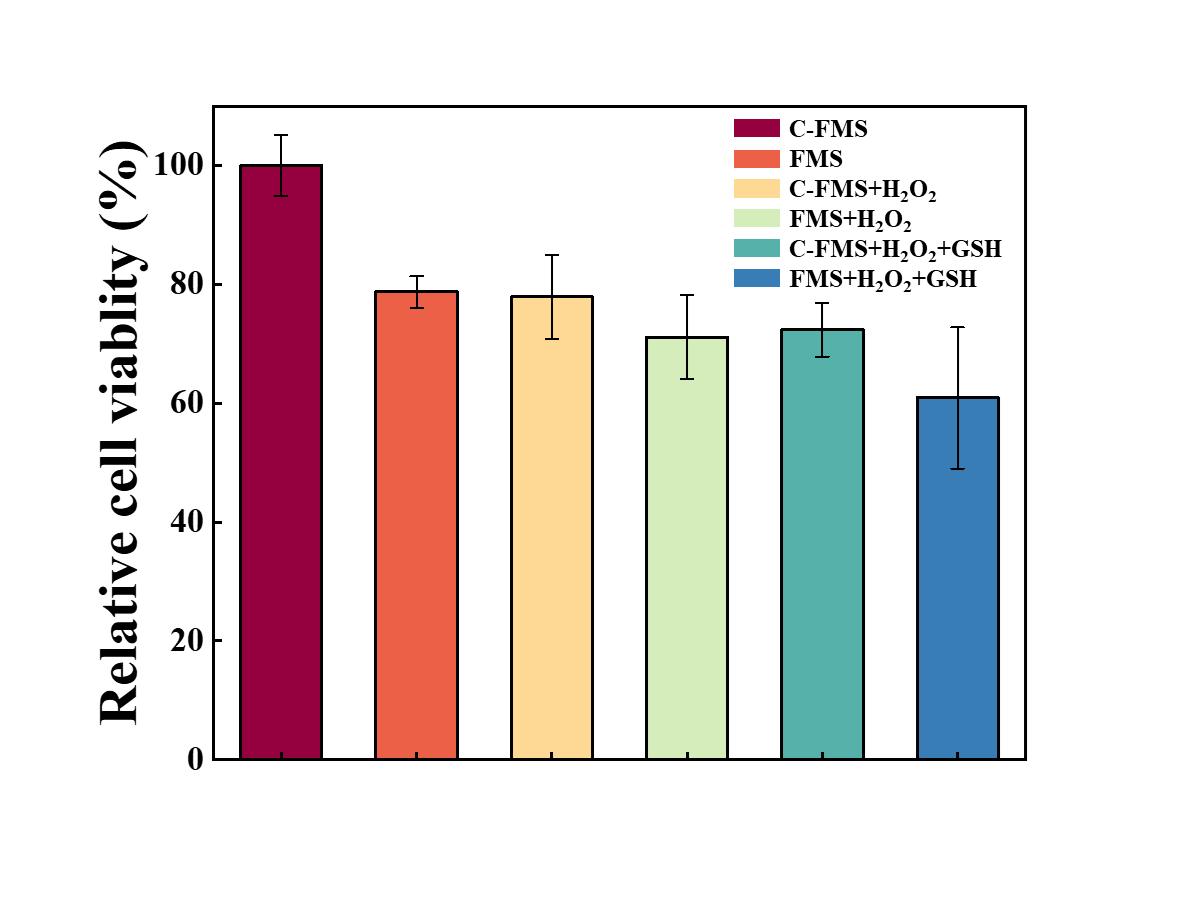


**Supplementary Figure 35.** Relative cell viability of 4T1 cells after co-incubation with C-FMS/FMS with or without H_2_O_2_ and GSH (in which C-FMS means core@shell structured FeOOH@mSiO_2_ nanoparticles, the data was normalized with C-FMS group as 100%). pH: 5.4. All conditions were fixed as experimental section mentioned above except for the alter one. The dosage of FMS and C-FMS in the *in vitro* experiments was all controlled at 200 μg/mL. The concentration of H_2_O_2_ and GSH in the *in vitro* experiments was all controlled at 10 mM. Data are expressed as mean standard ± errors (n = 3).

**
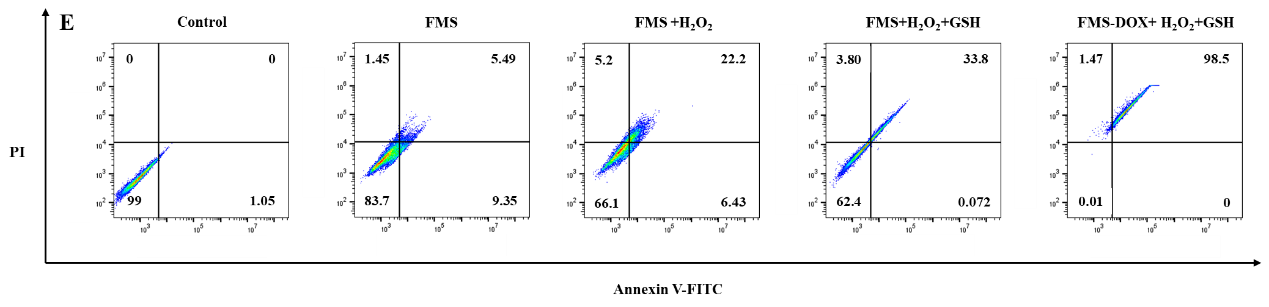
**

**Supplementary Figure 36.** Flow cytometric quantitative analysis of Annexin V-FITC/PI co-stained 4T1 cells after co-incubation with FMS/FMS-DOX with or without H_2_O_2_ and GSH under weak acidic (pH 5.4) conditions for 6 h. The dosage of FMS and FMS-DOX in the *in vitro* experiments was all controlled at 200 μg/mL. The concentration of H_2_O_2_ and GSH in the *in vitro* experiments was all controlled at 10 mM. No evident cell necrosis was observed.


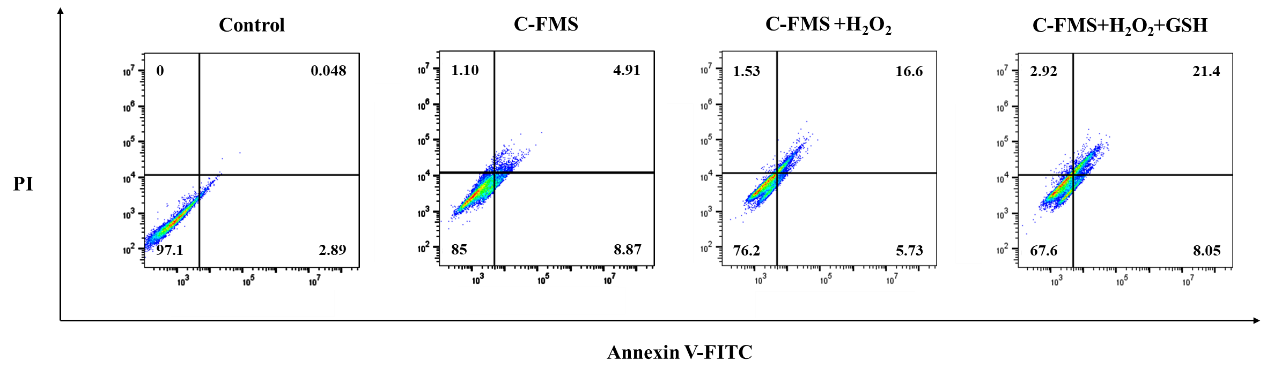


**Supplementary Figure 37.** Flow cytometric quantitative analysis of Annexin V-FITC/PI co-stained 4T1 cells after co-incubation with 200 μg/mL C-FMS with or without 10 mM H_2_O_2_ and GSH under weak acidic (pH 5.4) conditions. The dosage of C-FMS in the in vitro experiments was all controlled at 200 μg/mL. The concentration of H_2_O_2_ and GSH in the *in vitro* experiments was all controlled at 10 mM.

**
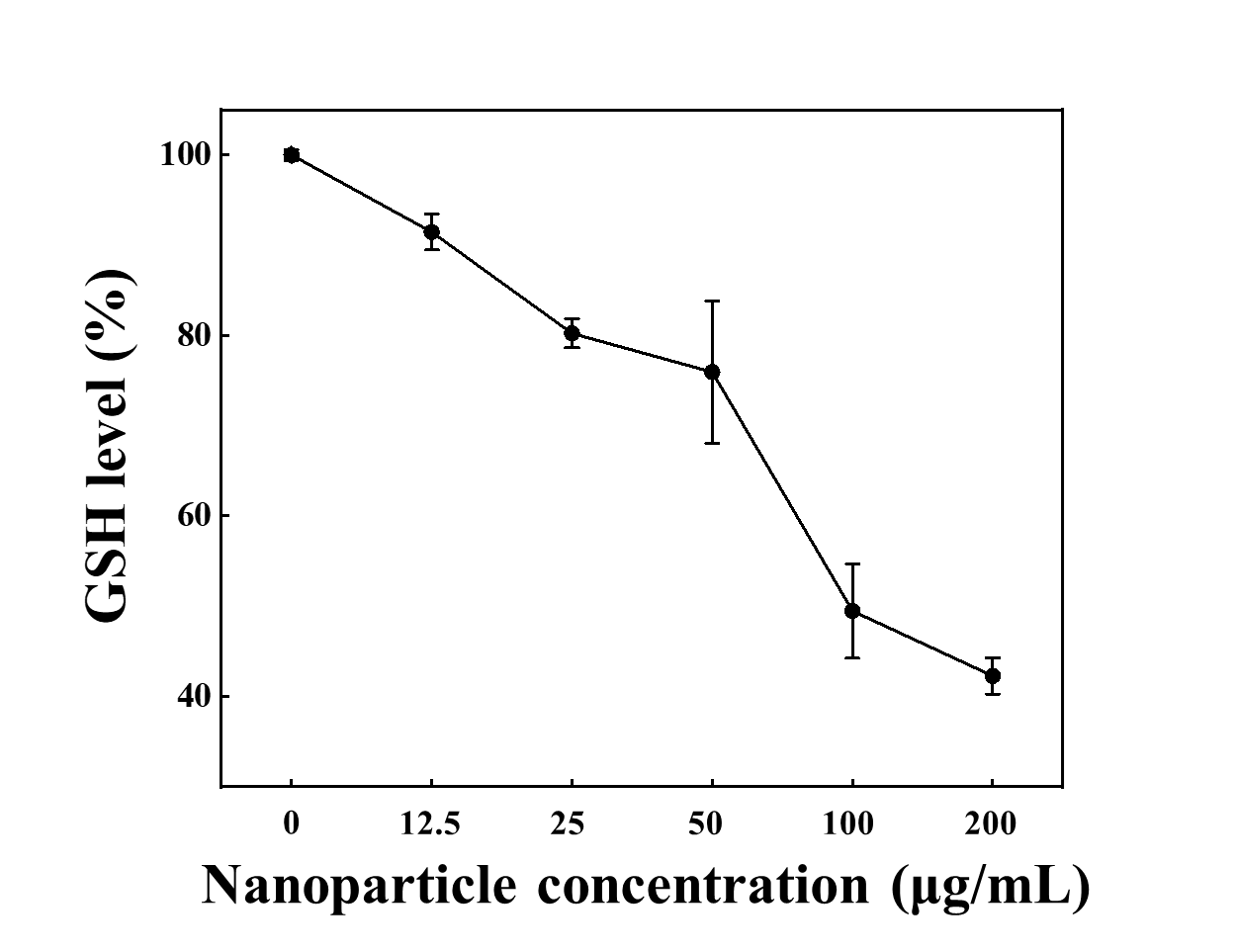
**

**Supplementary Figure 38.** Intracellular GSH consumption profiles monitored by UV/vis spectra at 412 nm with DTNB as a probe. Data is expressed as mean standard ± errors (n = 3).

**
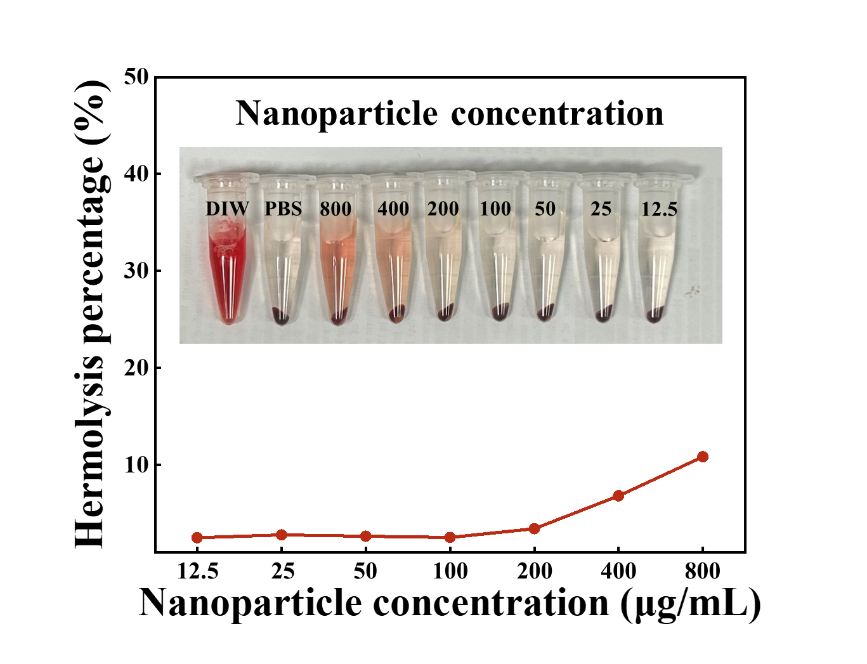
**

**Supplementary Figure 39.** The hemolysis analysis of sushi-like Janus FMS nanoparticles in the blood (mean ± SD, n = 3). Deionized water and PBS were used as positive and negative controls, respectively. The concentrations were determined by mass concentration of nanocomposites. It can be seen that all samples show negligible hemolytic effect even exposed to a high concentration of 400 μg/mL, indicating the superior hemo-compatibility of prepared nanoparticles.

**
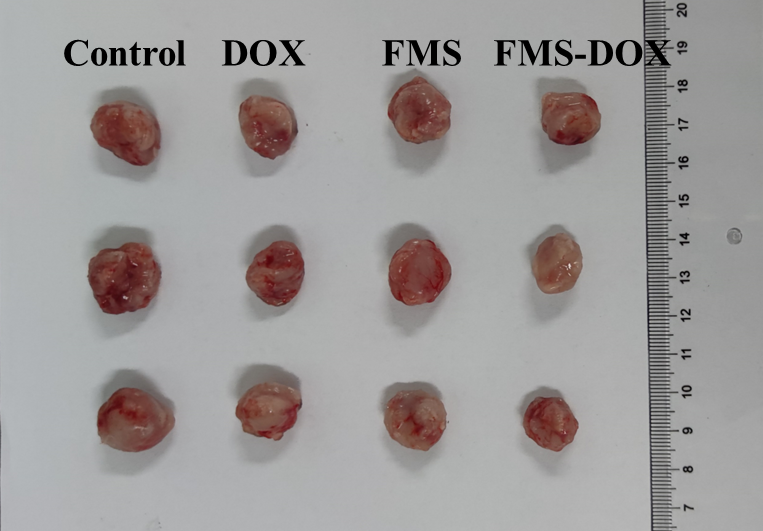
**

**Supplementary Figure 40.** Optical photograph of 4T1 tumors excised from four groups of 4T1-tumor-bearing mice treated with saline (control group), FMS, DOX and FMS-DOX (3 samples in each group).

**
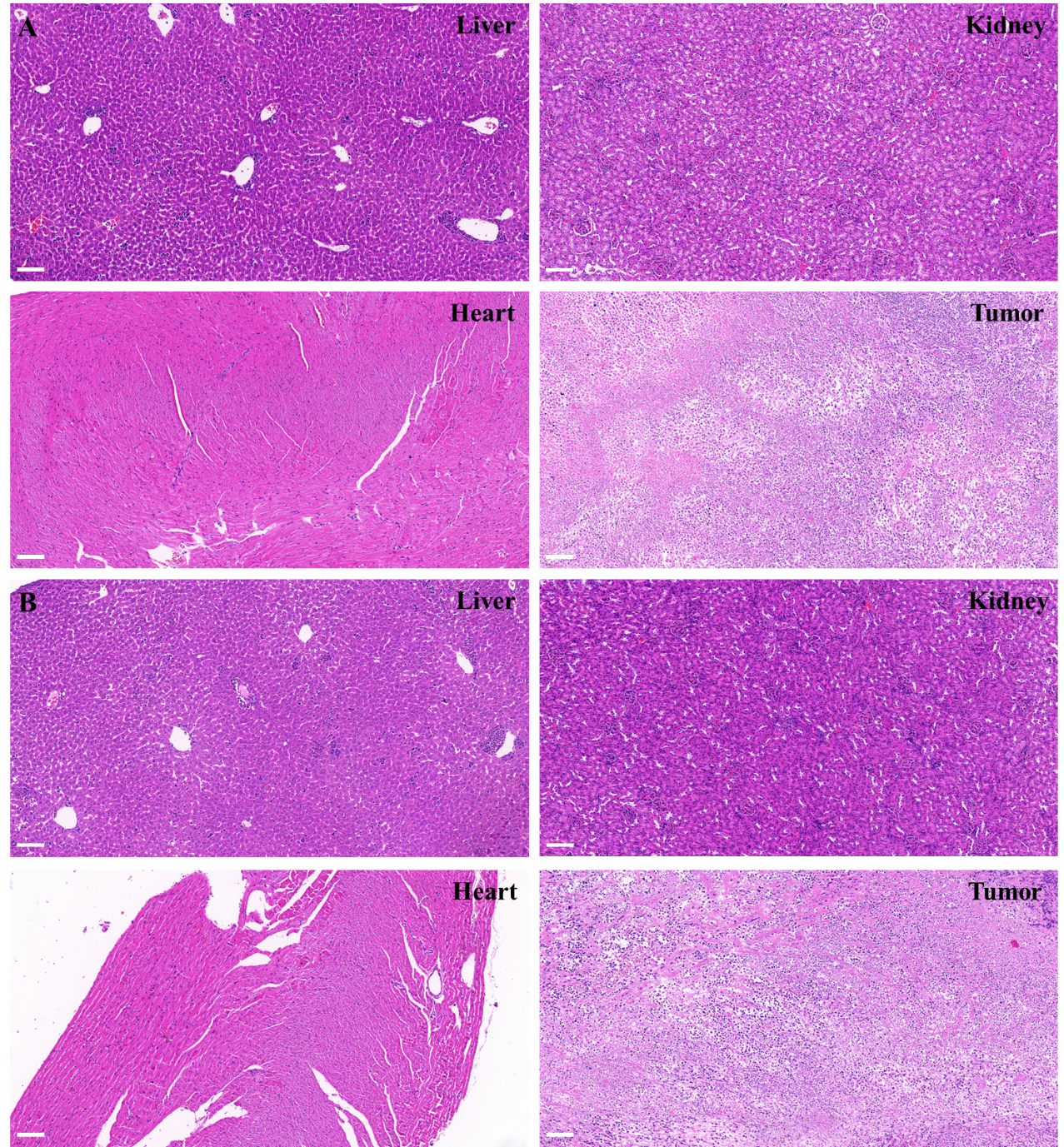
**

**Supplementary Figure 41.** H&E-stained tissue slice of liver, kidney, heart and tumor from the group treated with (A) Saline and (B) FMS+DOX, demonstrating that the nanoparticles do not damage no-lesion organs.

# Supplementary References

(1) Wang, X.; Chen, X.; Gao, L.; Zheng, H.; Ji, M.; Tang, C.; Shen, T.; Zhang, Z. Synthesis of β-FeOOH and α-Fe_2_O_3_ Nanorods and Electrochemical Properties of β-FeOOH. *Journal of Materials Chemistry* **2004**, *14* (5), 905–907.

(2) Deng, Y.; Qi, D.; Deng, C.; Zhang, X.; Zhao, D. Superparamagnetic High-Magnetization Microspheres with an Fe_3_O_4_@SiO_2_ Core and Perpendicularly Aligned Mesoporous SiO2 Shell for Removal of Microcystins. *Journal of the American Chemical Society* **2008**, *130* (1), 28–29.

(3) Li, X.; Zhou, L.; Wei, Y.; El-Toni, A. M.; Zhang, F.; Zhao, D. Anisotropic Growth-Induced Synthesis of Dual-Compartment Janus Mesoporous Silica Nanoparticles for Bimodal Triggered Drugs Delivery. *Journal of the American Chemical Society* **2014**, *136* (42), 15086–15092.

(4) Ye, X.; Zheng, C.; Chen, J.; Gao, Y.; Murray, C. B. Using Binary Surfactant Mixtures To Simultaneously Improve the Dimensional Tunability and Monodispersity in the Seeded Growth of Gold Nanorods. *Nano Letters* **2013**, *13* (2), 765–771.

(5) Li, L.; Lu, Y.; Jiang, C.; Zhu, Y.; Yang, X.; Hu, X.; Lin, Z.; Zhang, Y.; Peng, M.; Xia, H.; Mao, C. Actively Targeted Deep Tissue Imaging and Photothermal-Chemo Therapy of Breast Cancer by Antibody-Functionalized Drug-Loaded X-Ray-Responsive Bismuth Sulfide@Mesoporous Silica Core–Shell Nanoparticles. *Advanced Functional Materials* **2018**, *28* (5), 1704623.
